# High-dimensional profiling reveals Tc17 cell enrichment in active Crohn’s disease and identifies a potentially targetable signature

**DOI:** 10.1038/s41467-022-31229-z

**Published:** 2022-06-27

**Authors:** A.-M. Globig, A. V. Hipp, P. Otto-Mora, M. Heeg, L. S. Mayer, S. Ehl, H. Schwacha, M. Bewtra, V. Tomov, R. Thimme, P. Hasselblatt, B. Bengsch

**Affiliations:** 1 Department of Medicine II, Gastroenterology, Hepatology, Endocrinology, and Infectious Diseases, Faculty of Medicine, University Medical Center Freiburg, Freiburg, Germany; 2 Institute for Immunodeficiency, Center for Chronic Immunodeficiency, Faculty of Medicine, University Medical Center Freiburg, Freiburg, Germany; 3 Signalling Research Centres BIOSS and CIBSS, University of Freiburg, Freiburg, Germany; 4 German Cancer Consortium (DKTK), Heidelberg, Germany; 5 Department of Medicine, Division of Gastroenterology, University of Pennsylvania Perelman School of Medicine, Philadelphia, PA USA

**Keywords:** Lymphocyte differentiation, Inflammatory diseases, Mucosal immunology, Translational immunology, Crohn's disease

## Abstract

The immune-pathology in Crohn’s disease is linked to dysregulated CD4+ T cell responses biased towards pathogenic TH17 cells. However, the role of CD8+ T cells able to produce IL-17 (Tc17 cells) remains unclear. Here we characterize the peripheral blood and intestinal tissue of Crohn’s disease patients (n = 61) with flow and mass cytometry and reveal a strong increase of Tc17 cells in active disease, mainly due to induction of conventional T cells. Mass cytometry shows that Tc17 cells express a distinct immune signature (CD6^high^, CD39, CD69, PD-1, CD27^low^) which was validated in an independent patient cohort. This signature stratifies patients into groups with distinct flare-free survival associated with differential CD6 expression. Targeting of CD6 in vitro reduces IL-17, IFN-γ and TNF production. These results identify a distinct Tc17 cell population in Crohn’s disease with proinflammatory features linked to disease activity. The Tc17 signature informs clinical outcomes and may guide personalized treatment decisions.

## Abstract (web summary)

The T cell compartment in patients with Crohn's disease is dysregulated. Here the authors use cytometric profiling to reveal an enrichment of distinct Tc17 cells during active Crohn's disease and may suggest CD6 as a potential target for therapeutic studies.

## Introduction

Crohn’s disease (CD) is a currently incurable immune-mediated chronic inflammatory bowel disease (IBD) that can manifest in all areas of the gastrointestinal tract and causes significant morbidity in affected patients. Despite recent advances in immunosuppressive therapies, there is a large unmet need for better therapeutic options. CD is thought to be driven by T helper cell responses towards luminal antigens triggered by breakdown of mucosal immune homeostasis in genetically predisposed individuals^1–3^. Pathogenic CD4+ T cell responses with features of both TH17 cells and TH1 cells with the ability to produce the inflammatory cytokines IL-17, IFN-γ and TNF have been linked to active inflammation in patients with CD and ulcerative colitis (UC)^4–10^. However, despite the frequent involvement of CD8+ T cells in tissue damage in other autoimmune-mediated diseases^11^, the role of CD8+ T cells in IBD is less well defined. Notably, in addition to IL-17 production by “conventional” CD8+ T cells, which recognize peptide antigens presented by MHC molecules, several other, “unconventional”, CD8+ T cell populations that can produce IL-17 (“Tc17” cells) have been described, such as γδ T cells, Mucosa-associated invariant T cells (MAIT) or Natural Killer T cells (NKT) that can recognize non-canonical TCR ligands of possible microbial origin, including lipid antigens, or metabolites derived from mevalonate or riboflavin biosynthetic pathways^12–17^. The frequencies of these Tc17 cells in the peripheral blood are typically low, whereas Tc17 cell frequencies in the intestine are higher^18^, in agreement with a role in maintaining mucosal immune homeostasis. Similar to TH17 cells, the differentiation of Tc17 cells is IL-6, TGF-β and STAT3-dependent^19^. Assessment of Tc17 cells is most precisely performed by cytokine staining requiring prior stimulation. Phenotypically, expression of C-type lectin-like receptor CD161 and high levels of dipeptidylpeptidase IV (CD26) were reported as markers of Tc17 cells, but these molecules can also be expressed by non-Tc17 cells^20–23^. Mass cytometric profiling has expanded the number of markers that can be interrogated simultaneously for single-cell profiling to >40 and has helped to map the mucosal immune system or to identify systemic and local immune signatures that characterize inflammatory bowel diseases^24–26^. However, comprehensive phenotypic profiling of Tc17 cells in IBD is lacking.

We hypothesized that dysregulation of Tc17 cells contributes to disease activity in CD and that immune signatures of Tc17 cells could represent innovative biomarkers and therapeutic targets. To determine the role of Tc17 cells in the peripheral blood and intestinal tissue of prospectively recruited CD patients, we therefore performed a comprehensive immunoprofiling of CD8+ T cells using flow cytometry and high-parametric mass cytometry. We identified Tc17 cells as enriched in active disease in the peripheral blood and intestinal tissue. High-dimensional analysis revealed a characteristic surface expression profile of these disease-associated cells. A transcriptomic signature based on the expression profile of Tc17 cells was associated with flare-free patient survival in a post hoc analysis of a prospectively recruited cohort of CD patients. We demonstrate that signature-based targeting of CD6 with an anti-CD6 antibody in clinical use to treat psoriasis targets Tc17 signature cells and reduces IL-17 production as well as IFN-γ and TNF production. These data shed light on the role of Tc17 cells in IBD and suggest that these cells represent attractive biomarkers and potentially promising therapeutic targets.

## Results

### IL-17 producing CD8+ T cells are enriched in active disease

**Fig. 1:**
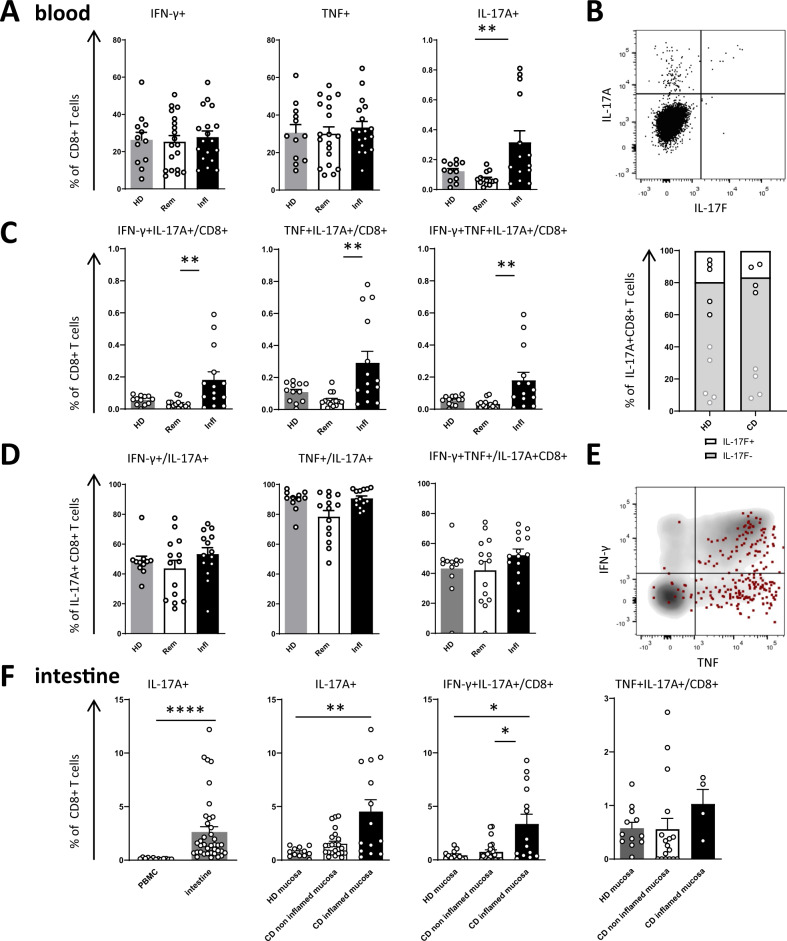
IL-17 production by CD8+ T cells is increased in active CD. **A** Production and co-production (**C**) of IFN-γ, TNF and IL-17 by CD8+ T cells isolated from the peripheral blood was compared between healthy donors (HD, *n* = 12) and CD patients in remission (Rem, *n* = 19) or with active disease (Infl, *n* = 18). **B** Representative flow cytometry plot depicting IL-17A and IL-17F production of CD8+ T cells isolated from the peripheral blood of a CD patient with active disease and bar graph depicting IL-17F coproduction of IL-17A producing CD8+ T cells isolated from the blood of HD (*n* = 5) and CD (*n* = 4). **D** Analysis of IFN-γ and TNF co-production by Tc17 cells (HD, *n* = 11; Rem, *n* = 14; Infl, *n* = 14). **E** Overlay density plot depicts IFN-γ and TNF production by IL-17 producing (red dots) CD8+ T cells from the peripheral blood of a representative CD patient with active disease. **F** Comparison of IL-17 production in peripheral blood and intestinal mucosa of CD patients (*n* = 38). Production of IL-17 and co-production of IFN-γ, IL-17 or TNF by CD8+ T cells in the intestinal mucosa was analyzed based on inflammation. Healthy donors (HD, *n* = 12), CD patients: non-inflamed (*n* = 24) and inflamed (*n* = 14) intestinal mucosa. Data in A, C, D, F are presented as mean +/− SEM. Statistical tests used were two-sided. ****p < 0.0001, ***p < 0.001, **p < 0.01, *p < 0.05.

Despite a prominent role for polarized TH1/TH17 CD4+ T helper cell responses in active IBD and in particular in CD^2–8^, the role of polarized CD8+ T cell responses is poorly understood in these disease entities. We therefore analyzed CD8+ T cells from CD patients with active disease and in remission stimulated for production of type 1 cytokines IFN-γ and TNF, as well as type 17 cytokines IL-17A and IL-17F. Interestingly, this analysis revealed a significant increase in IL-17A producing CD8+ T cells (Tc17 cells) in CD patients with active disease, while the percentage of CD8+ T cells expressing IFN-γ and TNF remained unchanged (Fig. 1A). Analysis of IL-17A and IL-17F (co)production revealed that the vast majority of Tc17 cells produced IL-17A, with minimal production of IL-17F that was coproduced by ~20% of IL-17A – producing CD8+ T cells (Fig. 1B, Supplementary Fig. 1). IL-17A will be subsequently referred to as IL-17 in this manuscript. Polyfunctionality analysis demonstrated that Tc17 cells co-expressing IFN-γ and TNF were significantly induced in active CD, highlighting their pro-inflammatory cytokine profile (Fig. 1C). Interestingly, this was due to the enhanced frequency of Tc17 cells, and not due to changes in their co-expression profile (Fig. 1D). Notably, Tc17 cells preferentially co-produced TNF (91% positive) while 53% co-produced IFN-γ, with 52% of Tc17 cells being triple producers of these inflammatory cytokines in active disease (Fig. 1D, E). The preference to co-produce TNF over IFN-γ was conserved between active CD, remission and healthy individuals, suggesting that it is intrinsically linked to the Tc17 program (Fig. 1D, E). These results demonstrate the induction of pro-inflammatory Tc17 cells with features of both type 1 and type 17 immunity in CD patients with active disease.

Patients had a diverse treatment history, with most patients treated with anti-TNF antibodies reflecting treatment guidelines (see Supplementary Tables 2–4); however, in multivariate analysis, Tc17 frequencies were only significantly linked to the inflammatory activity (*p* = 0.007) rather than different treatment history (*p* = 0.899).

We speculated that the enrichment of Tc17 cells in the peripheral blood reflected changes in intestinal mucosal immunity at sites of active disease. Indeed, analysis of CD8+ T cell function in intestinal biopsies revealed an even stronger induction of Tc17 responses with co-production of pro-inflammatory cytokines TNF and IFN-γ in the intestinal mucosa (Fig. 1F). The enrichment of Tc17 responses was observed at mucosal sites with endoscopic signs of inflammation, but was absent in non-inflamed mucosa, pointing towards the involvement of pro-inflammatory Tc17 cells in mucosal damage (Fig. 1F).

### High-dimensional analysis reveals polyfunctional Tc17 cells

**Fig. 2:**
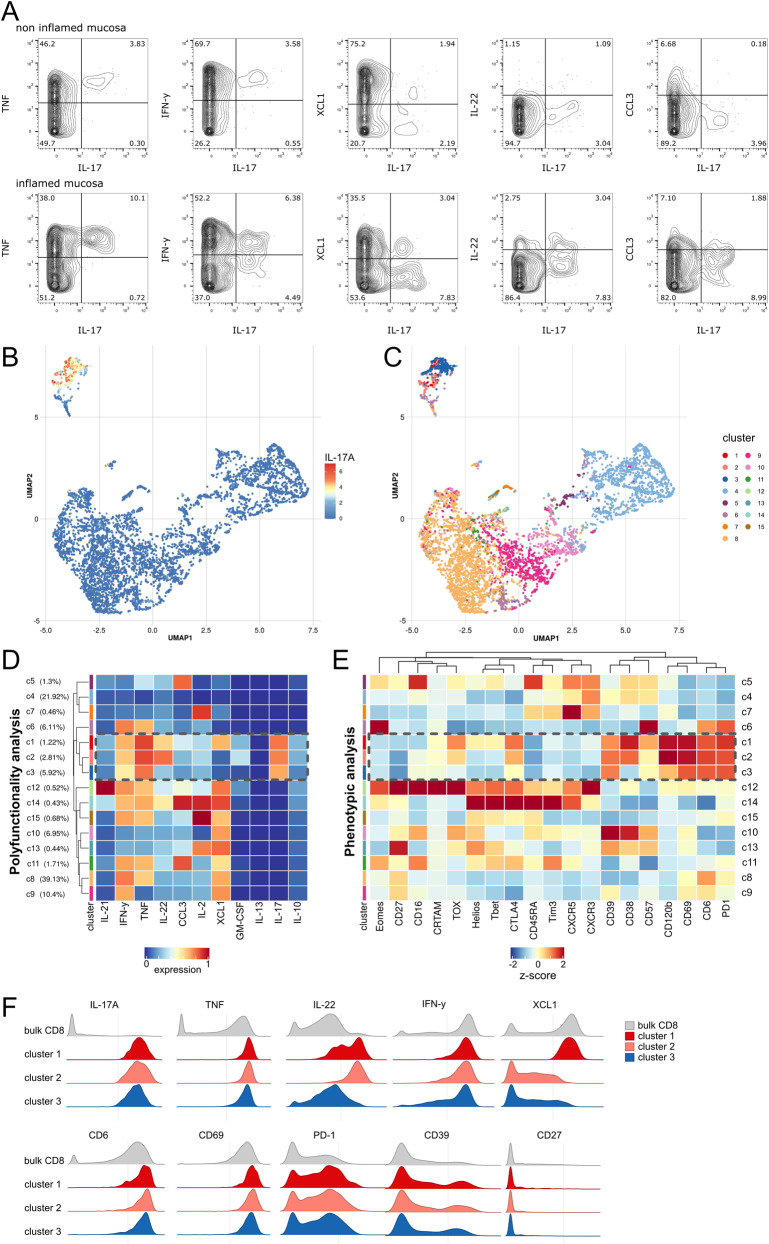
High dimensional mass cytometry analysis reveals a signature of IL-17 producing CD8+ T cells. **A** Representative mass cytometry plots gated on CD8+ T cells depict production of TNF, IFN-γ, XCL1, IL-22 and CCL3 plotted versus IL-17 of live singlet CD45+ CD3+ CD8+ T cells in the intestinal mucosa of a CD patient with proximal inflammation (*n* = 1646 and *n* = 690 CD8+ T cells in non-inflamed vs. inflamed mucosa, respectively). **B** UMAP analysis and FlowSOM clustering of the functional CD8+ T cell landscape from 6 CD biopsies were calculated on total *n* = 27768 CD8+ T cells based on IL-21, IFN-γ, TNF, IL-22, CCL3, IL-2, XCL1, GM-CSF, IL-13, IL-17 and IL-10 expression. IL-17 expression (**B**) and FlowSOM clusters (**C**) are indicated by color scheme. **D** Functional characteristics of FlowSOM clusters of intestinal CD8+ T cells are indicated by heatmap. Color scheme depicts arcsinh-transformed expression values scaled between 0.01 and 0.99 quantile. Hierarchical clustering was performed on unscaled data. Dashed box indicates the Tc17 clusters. **E** FlowSOM clusters were manually gated for expression of phenotypic markers and hierarchical clustering was performed on expression data. Frequency of positive populations per cluster is displayed by *z* score. **F** Histograms depict functional (top) and phenotypic (bottom) markers of clusters c1, c2 and c3 and bulk CD8+ T cells.

The finding of a strong and specific induction of Tc17 cells in the inflamed mucosa and peripheral blood of CD patients with active disease prompted us to further evaluate whether disease-associated polyfunctional Tc17 cells could serve as innovative biomarkers or therapeutic targets in active CD. First, we wanted to determine whether the observed polyfunctionality of Tc17 cells with respect to type 1 and type 17 cytokines extended to additional T cell cytokines and chemokines. Moreover, we sought to identify phenotypic markers of this population. To address these important questions, we designed a high-parametric mass cytometry panel that enabled us to comprehensively dissect the polyfunctionality and phenotype of intestinal Tc17 cells in IBD patients (Supplementary Table 1). To determine the possible intestinal heterogeneity of Tc17 phenotypes in CD, we obtained mucosal intestinal samples from CD patients with varying disease activity that were then stimulated and assessed for co-production of cytokines and chemokines using mass cytometry (Fig. 2A and Supplementary Fig. 2). The data were subsequently analyzed using FlowSOM clustering and uniform Manifold Approximation and Projection for dimension reduction (UMAP)^27^ to obtain insights into the polyfunctionality expression patterns linked to Tc17 cells. UMAP analysis based on 11 cytokines/chemokines (IL-21, IFN-γ, TNF, IL-22, CCL3, IL-2, XCL1, GM-CSF, IL-13, IL-17 and IL-10) revealed Tc17 cells as a distinct population of cytokine-producing CD8+ T cells (Fig. 2B). FlowSOM clustering analysis further identified 15 T cell clusters with distinct polyfunctionality patterns, of which three T cell clusters consisted of Tc17 cells (Fig. 2C–F, Supplementary Fig. 2). These Tc17 clusters (clusters c1, c2 and c3) displayed high co-production of TNF, while only two clusters (c1 and c2) showed high IFN-γ co-production, in agreement with the high levels of TNF co-production by Tc17 cells observed in our flow cytometry experiments (Figs. 1D, 2D). The highest polyfunctionality of all Tc17 clusters was observed in c1 that also displayed prominent expression of the chemokine XCL-1, while cluster c2 highly co-expressed IL-22 (Fig. 2D). Interestingly, these Tc17 cell clusters did not show evidence of type 2 immunity bias (absence of IL-13 production), and poorly expressed type 17 related cytokine GM-CSF. Moreover, only low expression of the immunoregulatory cytokine IL-10 could be identified in these Tc17 cells (Fig. 2D).

Together, these results indicate that intestinal disease-associated Tc17 cells represent a distinct population of inflammatory CD8+ T cells prone to co-produce TNF that can be further functionally subdivided into three subpopulations based on IFN-γ, IL-22 and XCL-1 production.

### High-dimensional analysis reveals a distinct Tc17 signature

We next sought to understand the phenotype of intestinal Tc17 cells by determining the expression patterns of a large panel of molecules involved in the activation, regulation, transcriptional programming and differentiation of CD8+ T cell responses on the functionally defined high-dimensional clusters. This approach based on our functional definition of CD8+ T cell clusters (Fig. 2D) is informative about the phenotypic heterogeneity of several functionally diverse CD8+ T cell populations in IBD (Fig. 2D–F). Here, we concentrated on understanding specific expression patterns of Tc17 cells and found that c1, c2 and c3 share high expression of molecules such as CD6, CD39, CD69, CD120b and PD-1 with a low expression of CD27 (Fig. 2D–F). Analysis of the transcriptional regulators T-bet, Eomes and Helios revealed rather low expression in Tc17 cells. Only in c1, the cluster with highest Tc17 polyfunctionality, some levels of T-bet expression could be observed (Fig. 2D–F), in agreement with the established role of T-bet in promoting type 1 responses in TH17 cells^4^. Moreover, some expression of the transcription factor Tox that is frequently observed in cells exposed to persistent antigen stimulation and chronic inflammation was found in Tc17 clusters c1 and c2, but not Tc17 cluster c3. These analyses suggested a role for T-bet and Tox in specifying the cytokine programs of Tc17 cells, but also indicated that other transcription factors determine the broader Tc17 differentiation program as master regulators. Indeed, we observed high and specific expression of retinoid acid receptor-related orphan receptor (ROR) γt on Tc17 cells (Supplementary Fig. 3). Of note, our approach also identified other clusters with expression patterns linked to known conventional CD8+ T cell differentiation states in addition to Tc17 cells. For example, cluster c12 and c14 expressed high levels of T-bet, IFN-γ and TNF indicating features of Tc1 cells while cluster c6 expressed high levels of Eomes and PD-1, resembling exhausted T cells. Of note, some markers highly expressed by Tc17 cells, such as CD6, were also expressed by other cell subsets, but the combination of CD6, CD39, CD69, CD120b and PD-1 with a low expression of CD27 was not observed on non-Tc17 cells. In sum, using this high-dimensional mass cytometric single-cell profiling approach, we identified a phenotypic signature of polyfunctional intestinal Tc17 cells linked to disease activity in CD patients.

### Validation of the Tc17 signature in an independent cohort

**Fig. 3:**
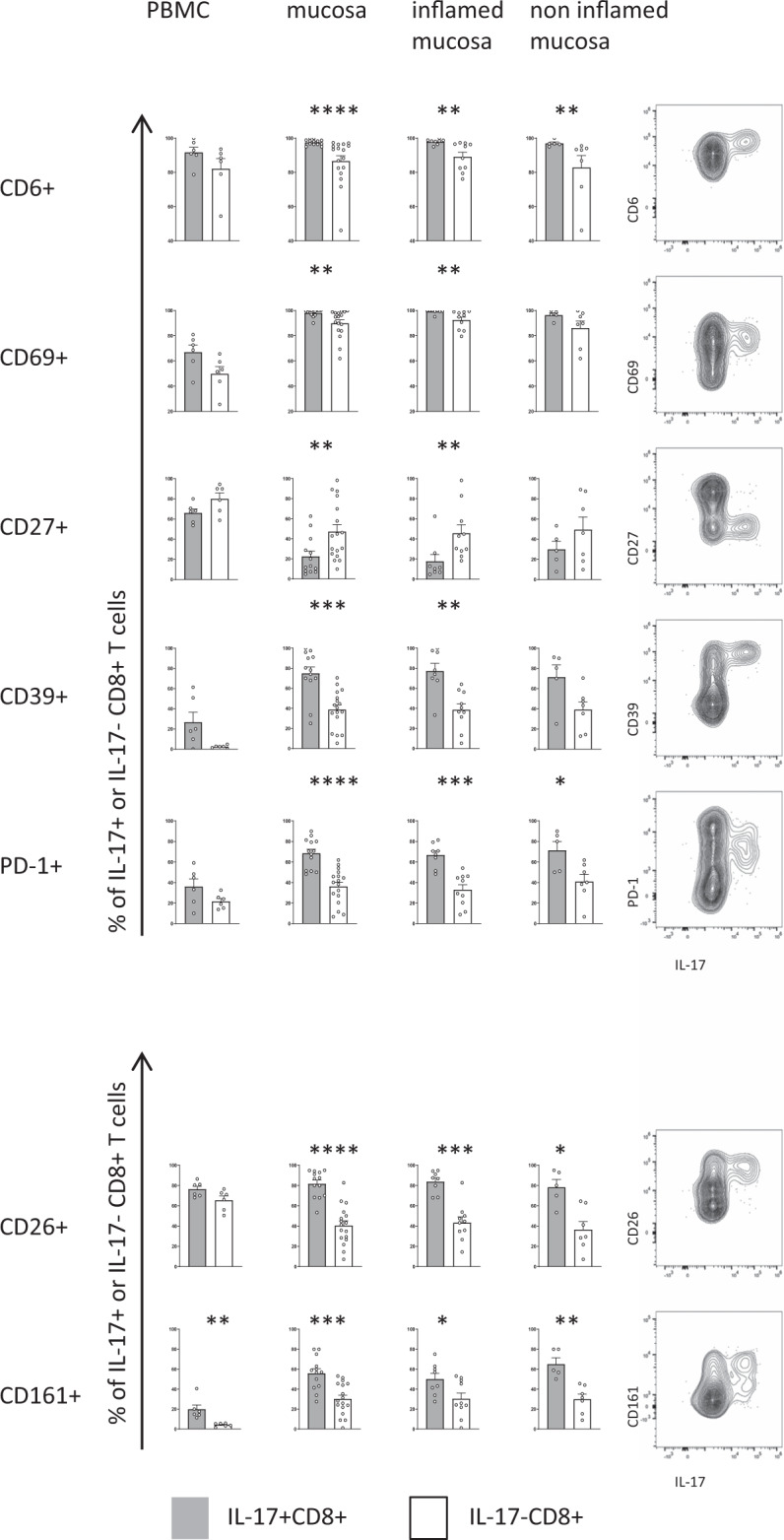
Flow cytometric validation of the Tc17 signature in an independent cohort of CD patients. The expression of phenotypic IL-17 signature markers in CD8+ T cells after PMA/ionomycin stimulation was compared between IL-17 producing (grey) and non-IL-17 producing CD8+ T cells (white) from peripheral blood (*n* = 6) and intestinal mucosa (*n* = 17) and analyzed based on endoscopic inflammation: inflamed mucosa (*n* = 10) and non-inflamed mucosa (*n* = 7). (Right) Representative FACS plots of signature markers versus IL-17 production by CD8+ T cells isolated from an inflamed mucosal sample of a CD patient. Data are presented as mean +/− SEM. Statistical tests used were two-sided. ****p < 0.0001, ***p < 0.001, **p < 0.01, *p < 0.05.

We next sought to validate the identified signature of IL-17 producing CD8+ T cells in an independent cohort of CD patients using conventional flow cytometry and to test this signature together with markers described for the characterization of Tc17 cells in earlier studies, such as CD26 and CD161^12,20^. As predicted by the high-dimensional mass cytometry analysis (Fig. 2), flow cytometric analysis of IL-17 producing and non-producing CD8+ T cells from peripheral blood and intestinal samples of CD patients demonstrated that Tc17 cells express significantly more CD6, CD69, CD39 and PD-1, but less CD27 (Fig. 3). Interestingly, analysis of this signature on CD4+ T cells in the same cohort also revealed a higher expression of CD6 and CD69 on TH17 cells in the peripheral blood and intestinal mucosa, while the expression of CD27, PD-1 and CD39 was not clearly linked to the TH17 phenotype, in contrast to Tc17 cells (Supplementary Fig. 4). Moreover, both Tc17 and TH17 cells also expressed CD161 and high levels of CD26 (Fig. 3, Supplementary Fig. 4). In sum, the comparison of the Tc17 signature with TH17 cells points towards conserved expression of CD6 and CD69 but also relevant differences in the CD8+ Tc17 and CD4+ TH17 programs. Thus, these data validate and extend the disease-associated single-cell Tc17 signature in IBD patients.

### Most Tc17 cells in CD reflect conventional T cell responses

**Fig. 4:**
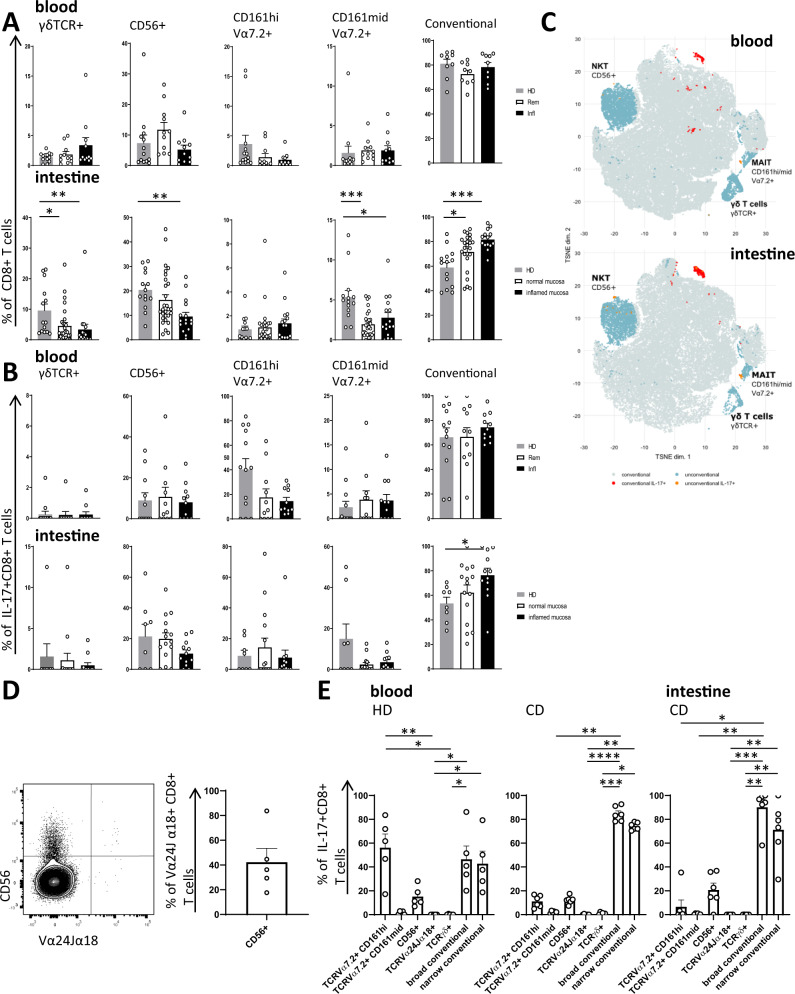
Contribution of unconventional T cell responses to IL-17 production in CD. Flow cytometry analysis of PMA/Ionomycin stimulated cells indicating percentage of γδ T cells (γδTCR+), NKT cells (CD56+ TCR γδ- Vα7.2-), MAIT cells (CD161^hi^ Vα7.2+ or CD161^mid^ Vα7.2+) and conventional T cells (TCR γδ- CD56- Vα7.2-) of CD8+ T cells (**A**) and of Tc17 cells (**B**). Comparison of indicated frequencies in PBMC as well as in the intestinal tissue of HD (**A** *n* = 18, **B** *n* = 12), non-inflamed mucosa from CD patients (normal mucosa, **A** *n* = 28, **B** *n* = 15) and inflamed mucosa from CD patients (inflamed mucosa, **A** *n* = 16, **B** *n* = 12). **C** TSNE analysis of conventional and unconventional T cells calculated based on the lineage markers described above from peripheral blood and intestinal biopsies (*n* = 10) of 7 different CD patients with active disease. IL-17 production by unconventional (orange dots) and conventional (red dots) T cells is shown. **D** Frequency of CD56+ cells of Vα24Jα18 (Clone 6B11)+ CD8+ iNKT cells (*n* = 5). **E** Percentage of MAIT cells (CD161^hi^ Vα7.2+ and CD161^mid^ Vα7.2+), iNKT cells (Vα24Jα18+), γδ T cells (γδTCR+) and conventional T cells (defined as Vα7.2-, Vα24Jα18-, γδTCR- and αβ TCR+) of IL-17+ CD8+ CD3+ cells were examined in PBMCs of HD (*n* = 5), CD patients (*n* = 6) and in intestinal biopsies of CD patients (n biopsies = 6). Broad and narrow conventional T cell frequencies were determined taking into account only the iNKT (Vα24Jα18-) vs. both the iNKT and the CD56+ NKT definition (Vα24Jα18-, CD56-), respectively. Data in A, B, D, E are presented as mean + /− SEM. Statistical tests used were two-sided. ****p < 0.0001, ***p < 0.001, **p < 0.01, *p < 0.05.

The ability to produce IL-17 has been reported for several CD8+ T cell populations, including conventional αβ T cells recognizing cognate peptide-MHC-I complexes by their TCR, but also various unconventional CD8+ T cell populations, e.g. γδ T cells, NKT cells and MAIT cells^12–17,28,29^. We therefore asked if the induction of Tc17 cells was due to an expansion of conventional or unconventional T cells and whether it reflected dynamics of conventional or unconventional T cell responses on the bulk CD8+ T cell level. Frequencies of γδ T cells, NKT cells and MAIT cells of CD8+ T cells and Tc17 cells were determined in peripheral blood and intestinal CD8+ T cell populations in patients with active CD and compared to inactive disease and healthy individuals (Fig.4, Supplementary Fig. 5). In the peripheral blood, in bulk CD8+ T cells, we observed higher frequencies of γδ T cells in patients with active inflammation, in line with other reports on the enrichment of γδ T cells in CD^30–33^ (Fig. 4A). However, analysis of the intestinal tissue revealed a major reduction of γδ CD8+ T cells (as well as NKT cells) in the inflamed mucosa while conventional CD8+ T cells were increased. Moreover, we did not observe a significant contribution of γδ T cells to IL-17 production, neither in the periphery nor the intestinal tissue (Fig. 4B, C). In contrast to the very low frequencies of IL-17-producing γδ CD8+ T cells, we observed that the majority (>65%) of IL-17 production by CD8+ T cells in active CD was by conventional T cells, with a minor contribution of MAIT or NKT cells (Fig. 4B, C). Since some CD1d-restricted invariant Vα24Jα18 TCR-expressing NKT cells may not strictly express CD56, and CD56 may also be expressed by other MAIT and conventional CD8+ T cells, we stained specifically for this iNKT cell population using 6B11 monoclonal antibody^34^. As expected, these iNKT cells showed variable CD56 expression (Fig. 4D). In comparison to other types of IL-17-producing CD8+ T cells, frequencies of iNKTs were negligible (Fig. 4E). While the reduction of γδ CD8+ T cells in the intestine and their infrequent IL-17 production were unexpected, these results could point to an efflux of γδ T cells from the intestine into the peripheral blood but also to an accumulation of polarized pro-inflammatory conventional T cells at the site of inflammation. A different pattern was observed for IL-17 producing CD161^hi^ Vα7.2+ CD8+ MAIT cells, which were reduced in the peripheral blood of CD patients but were found at similar frequency in the intestinal mucosa (Fig. 4B, E). Interestingly, in healthy individuals, MAIT cells represented a major source of IL-17 produced by CD8+ T cells in the peripheral blood, in particular in individuals with high frequencies of MAIT cells, but this MAIT-bias of Tc17 cells was not observed in the inflamed CD context, where we observed an emergence of conventional T cells as the major source of IL-17 (Fig. 4B, E). Moreover, we did not observe an expansion of CD161- Vα7.2+ CD8+ T cells in the inflamed intestinal mucosa, arguing against potential loss of CD161 or recruitment of conventional Vα7.2+ CD8+ T cells (Supplementary Fig. 6). Despite these major differences in conventional and unconventional CD8+ Tc17 dynamics, RORγt expression was similarly high on IL-17 producing cells across these T cell subtypes (Supplementary Fig. 3), indicating that the preferential induction of conventional over unconventional Tc17 responses in CD is not due to an inability to express the transcriptional master regulator of the type 17 program. In sum, these results identify conventional T cells as the major type of Tc17 cells induced in active CD.

### The Tc17 signature discriminates flare-free survival in CD

**Fig. 5:**
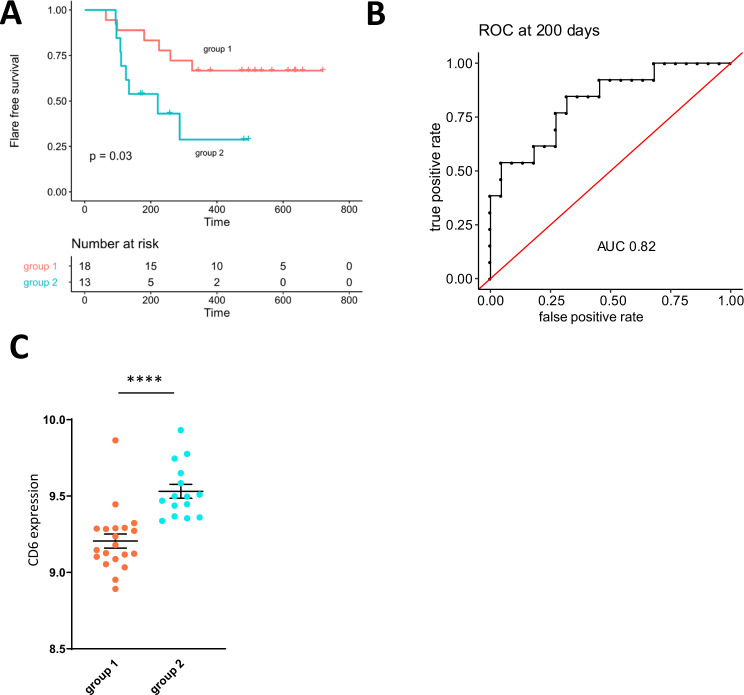
The Tc17 signature is associated with flare-free survival. **A** CD8+ T cell transcriptome data and corresponding clinical data from 31 untreated CD patients were extracted from E-MTAB-331^35^ and patients were stratified into two groups based on hierarchical clustering on phenotypic Tc17 signature markers CD6, CD69, CD27, CD39, PD-1, CD26, CD161. Kaplan-Meier-plot depicts flare-free survival. Log rank test was used to determine statistical significance. **B** Time dependent ROC curve for prediction of flare free survival at 200 days of follow up using a GSVA score based on phenotypic Tc17 signature markers CD6, CD69, CD27, CD39, PD-1, CD26, CD161 and CD8+ T cell transcriptome data and corresponding clinical data from 35 untreated CD patients that were extracted from^35^. **C** CD6 expression in group 1 and 2 indicated in arbitrary units, *n* = 35 patients extracted from E-MTAB-331. Data are presented as mean +/− SEM. Statistical tests used were two-sided. ****p < 0.0001, ***p < 0.001, **p < 0.01, *p < 0.05.

Currently, biomarkers to discriminate IBD patients with frequently relapsing disease from those with a more quiescent form of the disease are lacking. We sought to assess the predictive value of the Tc17 signature from our single-cell analysis in a published CD8+ T cell transcriptome dataset of untreated CD patients at initial diagnosis from Lee et al.^35^ that were followed up prospectively. For this analysis, 31 untreated CD patients were stratified into two groups of patients using unbiased hierarchical clustering based on gene expression corresponding to the phenotypic Tc17 signature markers identified by cytometry (*CD27, CD6, CD69, DPP4, ENTPD1, KLRB1, PDCD1*) and flare-free survival according to the clinical follow up data^35^ was determined. This approach identified significant differences in flare-free survival, indicating that differences in the Tc17 signature during inflammation are linked to important clinical outcomes (Fig. 5A, B, Supplementary Fig. 7). This analysis further underlines the relevance of Tc17 cells in IBD patients (Fig. 5A).

To validate the predictive value of our Tc17 signature for flare free survival further we performed a ROC analysis based on the GSVA score calculated using the identified Tc17 signature markers at a clinical endpoint of 200 days of follow-up. This analysis resulted in an AUC value of 0.82, highlighting the potential of our signature as a predictive marker for the clinical course of Crohn’s disease (Fig. 5B).

Notably, further subanalysis of the Tc17 signature showed that CD6, that had been identified as a highly expressed protein by disease-associated Tc17 cells in our high-dimensional single-cell profiling analysis (Fig. 2), also displayed the highest mRNA levels in those patients with reduced flare-free survival (Fig. 5C). We thus wondered whether the expression of CD6 by Tc17 cells could represent an opportunity for targeted interventions aiming to modulate Tc17 responses.

### Targeting CD6 reduces IL-17 production

**Fig. 6:**
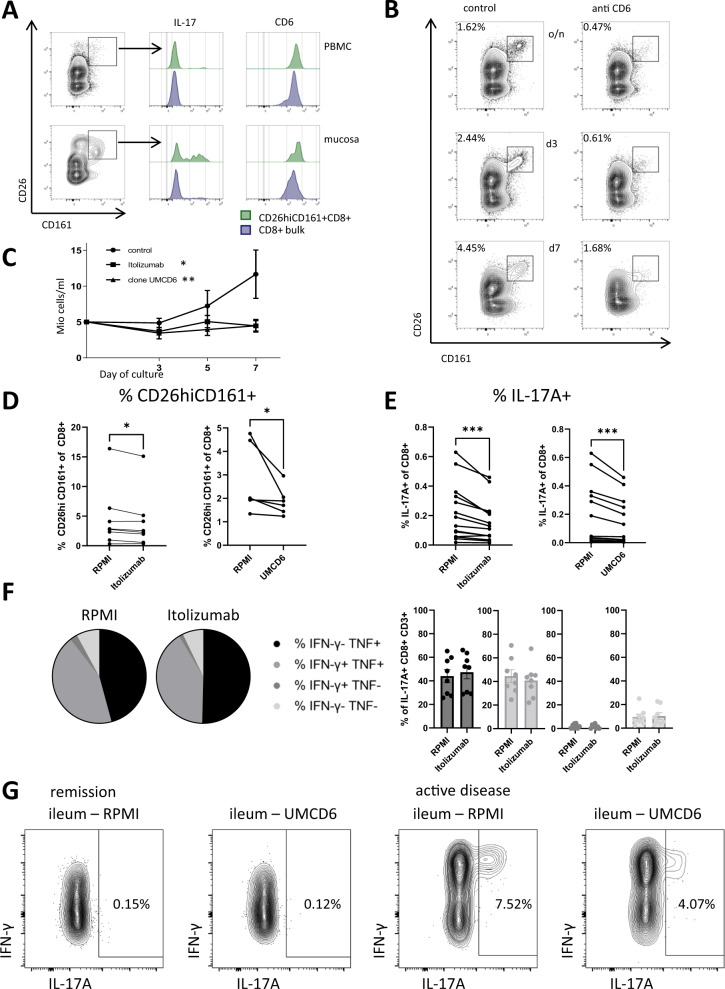
Targeting CD6 expressed by Tc17 cells reduces proinflammatory Tc17 cells. **A** CD26^hi^CD161+ CD8+ T cells contain Tc17 cells and express higher CD6 as illustrated by original plots and overlay histograms of live CD8+ and CD26^hi^CD161+ CD8+ T cells from PBMC and intestinal tissue of a CD patient analyzed for IL-17A and CD6. **B** Reduction in CD26^hi^CD161+ CD8+ T cells in presence of anti-CD6 (Itolizumab) treatment during culture of PBMCs overnight (o/n), or for three or seven days (d3, d7) (plots gated on CD8+ T cells). **C** Cell numbers in anti-CD6 vs. control-treated assays (*n* = 4 for each condition). **D** Percentage of CD26^hi^CD161+ CD8+ T cells in presence of RPMI, Itolizumab (*n* = 8) or UMCD6 (*n* = 5). **E** Percentage of IL-17A+ CD8+ T cells in presence of RPMI, Itolizumab (*n* = 14) or UMCD6 (*n* = 13). **F** Proportion of IFN-γ and TNF mono- and coproduction by IL-17A+ CD8+ CD3+ T cells in presence of RPMI or Itolizumab (*n* = 8). **G** IL-17A production of CD8+ T cells from intestinal biopsies after five-hour stimulation in presence of RPMI or UMCD6. Depicted are samples from a CD patient with clinical and endoscopic remission and a patient with clinical and endoscopic activity. **D**, **E** Data after 5 h PMA/Ionomycin stimulation; (**B**–**E**) assays performed on HD samples. Data in (**C**) and (**F**) are presented as mean +/− SEM. Statistical tests used were two-sided. ****p < 0.0001, ***p < 0.001, **p < 0.01, *p < 0.05.

The anti-CD6 antibody Itolizumab is clinically used to treat patients with psoriasis in Asia and has been proposed to alter T cell polarization programs by modulating intracellular signaling pathways controlled by CD6, as opposed to depleting CD6+ T cells^36–38^. We therefore hypothesized that targeting CD6 on Tc17 cells could functionally suppress this IBD disease-associated T cell population. Thus, we targeted CD6 by specific monoclonal antibodies (Itolizumab and clone UMCD6) in vitro in short term stimulation assays and under type 17 polarizing conditions. Anti-CD6 therapeutic antibodies rapidly bind to CD6 (Supplementary Fig. 8A). To minimize crosstalk of blocking and anti-CD6 staining antibodies, we first used CD26 and CD161 to identify Tc17 cells^20^. CD26^hi^CD161+ cells had the highest levels of CD6 expression (Fig. 6A). Exposure to anti-CD6 antibodies reduced CD26^hi^CD161+ cells after overnight addition and after culture for 3–7 days (Fig. 6B), indicating a major impact on type 17 polarized cells. Of note, these experiments also revealed a pronounced reduction of T cell expansion under anti-CD6 conditions (Fig. 6C, Supplementary Fig. 8B). We next asked whether a specific effect on Tc17 cells could be observed already after short term ex vivo exposure to anti-CD6. Tc17 polarization might be altered by CD6-mediated control of STAT3 signaling; however, we did not observe differences in STAT3 phosphorylation after IL-21 stimulation in short-term assays (Supplementary Fig. 9A). Strikingly, however, short-term stimulation of PBMC in the presence of anti-CD6 antibodies revealed a significant reduction in the frequency of CD26^hi^CD161+ CD8+ T cells and IL-17 production already after 5 h incubation (Fig. 6D, E, Supplementary Fig. 10). A similar suppressive effect of anti-CD6 was observed both on conventional and MAIT cells (Supplementary Fig. 8C).

### Anti-CD6 reduces proinflammatory Tc17 and TH17 cells

Since Tc17 cells in CD produce additional pro-inflammatory cytokines IFN-γ and TNF (Fig. 1), we next wondered whether anti-CD6 would also impact the effector cytokine co-production of Tc17 cells. Interestingly, we did not observe a change in the proinflammatory polyfunctionality profile of Tc17 cells after short term stimulation (Fig. 6F). However, there was also a net reduction of IFN-γ and TNF production by CD8+ T cells after anti-CD6 treatment, which exceeded the reduction of Tc17 cells (Supplementary Fig. 11). Of note, while we observed a small reduction in viability in anti-CD6 treated cultures, the observed reduction did not explain the magnitude of the anti-CD6 effects on cytokine production (Supplementary Fig. 11B). These data indicate that anti-CD6 reduces cytokine production by Tc17 cells but also additionally limits effector cytokine production in other CD8+ T cell subsets, in accordance with CD6 expression observed in non-Tc17 clusters (Fig. 2). We also tested for the effects of anti-CD6 on CD4+ T cells, since CD6 was also expressed on TH17 cells (Supplementary Fig. 4). Of note, similar to Tc17 cells, we observed a reduction of the CD26^hi^CD161+ CD4+ T cell subset, a reduction in IL-17 production by TH17 cells and an additional reduction of IFN-γ and TNF production in the CD4+ T cell compartment (Supplementary Fig. 12). Together, these data indicate that anti-CD6 limits the proinflammatory cytokine profile of Tc17 and TH17 subsets, but also reduces cytokine production in other effector T cells.

### Anti-CD6 reduces IL-17 in T cells from active CD lesions

We thus wondered if the anti-CD6 intervention would also reduce cytokine production in cells isolated from active CD lesions, in which pathogenic Tc17 cells are enriched (Fig. 1). Interestingly, short term culture of intestinal lymphocytes with anti-CD6 antibodies indeed revealed a reduction in IL-17 production in cells from a patient with active disease while it was less impactful on the low IL-17 production observed by intestinal lymphocytes isolated from a patient in remission (Fig. 6G). Interestingly, in cells from the patient with active disease, a reduction of TH17 cells was also observed (Supplementary Fig. 12). In sum, these experiments demonstrate that anti-CD6 antibodies can suppress Tc17 and TH17 responses and indicate a role for CD6 in regulating Tc17 function. They also inform that anti-CD6 has additional effects on non-type 17 T cells. Taken together, our data demonstrate an enrichment of conventional Tc17 cells with a proinflammatory cytokine profile in active CD, identify phenotypic markers for this Tc17 subset and highlight components of the Tc17 signature, such as CD6, as potential therapeutic targets in IBD.

## Discussion

This work identifies the functional and phenotypic profile of Tc17 cells as a disease-associated immune-cell population enriched in the peripheral blood and inflamed mucosa of patients with active CD. Deep immune profiling and algorithm-guided high dimensional data analysis revealed a signature of immune markers expressed by Tc17 cells that was validated in a separate cohort of CD patients using conventional flow cytometry. The signature we describe here allowed the identification of patients with different clinical outcomes in a retrospective transcriptome analysis. CD6 was part of the Tc17 signature highly expressed in CD and can be targeted by monoclonal antibodies which are already in clinical use to treat patients with psoriasis, providing a possible rationale for this therapeutic option in CD patients^36^. Anti-CD6 intervention in vitro reduced IL-17 production, but also pro-inflammatory IFN-γ and TNF production, indicating an overall anti-inflammatory effect on CD8+ T cells. The disease-associated Tc17 cells identified in this work have several similarities to polarized “pathogenic” CD4+ T cell responses that have features of both type 17 and type 1 immunity and are strongly enriched in several autoimmune mediated diseases, including CD^4,5,39^. In contrast to non-pathogenic TH17 cells, pathogenic TH17 cells are known to produce high levels of the effector cytokines TNF and IFN-γ that are implicated in severe inflammation and tissue damage. Notably, the Tc17 cells identified in this study as enriched in patients with active CD also had a high co-expression of TNF and IFN-γ, indicating similarities in effector function with the described pathogenic TH17 cells. Some markers that identify disease-associated Tc17 cells were also shared with TH17 cells, including CD6, CD26, CD69 and CD161, suggesting that these molecules could constitute attractive candidates for shared biomarker panels or targeted immunomodulatory approaches. Indeed, we also observed a reduction in the cytokine production profile of TH17 cells after anti-CD6 treatment in vitro.

The high-dimensional clustering analysis also identified a population of Tc17 cells that did not share the high IFN-γ expression (c3) we found enriched in active disease. Several intestinal CD8+ T cell populations with the ability to produce IL-17 have been reported and are implicated in maintenance of intestinal immune homeostasis, including γδ T cells, MAIT or NKT cells^13,28,29^. Interestingly, these unconventional T cell populations did not account for the strong enrichment of Tc17 cells observed in active disease, which was rather due to the induction of conventional T cell responses. This was unexpected since a clear trend towards enrichment of γδ T cells within the bulk CD8+ T cell compartment was observed in the peripheral blood of patients with high disease activity and has also been reported by others^31–33^. The significance of γδ T cell dynamics is further underlined by inclusion of γδ genes into the gene classifier in the PROFILE trial^30^. However, the reduction of γδ T cells in the intestinal mucosa during inflammation observed in our analysis suggests that these cells may not be directly driving inflammation. Clearly, the induction of Tc17 cells both in the peripheral blood and in the inflamed intestine did not reflect the dynamics of γδ T cells but rather the expansion of conventional CD8+ T cells. It is thus tempting to speculate that the induction of Tc17 cells in CD patients with high disease activity is linked to recognition of conventional peptide antigens. The identification of Tc17 cells in this work as a strongly disease-associated T cell population may therefore be helpful in studies aiming to identify disease-associated TCRs and antigens underlying the pathogenic immune responses driving CD.

MAIT cells are frequently considered to be the dominant source of CD8-derived IL-17. However, while we observed a MAIT bias with a contribution of about 50% to Tc17 responses in the peripheral blood in HD and patients in remission, this proportion was reduced in patients with active disease and in the intestine. The relative contribution of peripheral MAIT cell-derived IL-17 to the total Tc17 pool is also influenced by the baseline MAIT cell frequency, which itself is highly variable among individuals. Our data illustrates that MAIT cells do not always represent the dominant Tc17 population in humans and that Tc17 cells are heavily influenced by inflammatory disease activity.

Tc17 cells can in general produce both IL-17 cytokines IL-17A and IL-17F. Genetic ablation experiments in mouse models suggest that IL-17F can have a divergent effect on the microbiota in comparison to IL-17A and might be involved in mediating protection against colitis^40,41^. Previous publications on TH17 cells have suggested a large degree of co-expression of IL-17A and IL-17F^8^. Here, we found a clear bias towards IL-17A production in CD patients and controls, with ~20% of Tc17 cells co-producing IL-17A and IL-17F. These data indicate that the Tc17 response in CD is dominated by IL-17A, in accordance with a relative absence of the potentially protective IL-17F during inflammation. However, additional studies will be required to clarify the roles of IL-17A and IL-17F in CD, in particular with regard to quantification of other cellular sources and their targets.

Only small differences in Tc17 cells were observed between patients with inactive disease and healthy individuals. These data are in agreement with reports from a previous study that performed a focused analysis of Tc17 cells and regulatory T cells in UC and CD patients^18^. However, in that study, the role of Tc17 cells during inflammation remained unclear, likely due to the absence of patients with severe inflammation^18^. In our study, analysis of patients presenting with active CD analyzed with a deep immune profiling approach led to the identification of a clear link between pathogenic Tc17 cells and disease activity, indicating that these cells could serve as interesting biomarkers or therapeutic targets. This notion is supported by recent reports with single-cell atlas data highlighting an enrichment of Tc17 cells in the inflamed mucosa in IBD^26,42,43^.

The translational relevance of the disease-associated Tc17 responses is further underlined by two major aspects:

First, the finding that the Tc17 signature allows the stratification of patients with active disease into cohorts with different clinical outcomes, as shown by our analysis of the dataset published by Lee et al., which led to the identification of blood-based biomarker signatures that allow prediction of prognosis in newly diagnosed IBD patients^30,35^. Of note, the 7-marker signature for disease –associated Tc17 cells identified in this work based on mass cytometry analysis does not overlap with the 17-gene classifier used to predict prognosis in the PROFILE trial, yet also separated the patients with a complicated clinical course clearly^30^. Further studies will be required to test if the Tc17 cells identified here represent the cellular correlate reflected by the gene classifier or whether they represent an additional immune correlate informative of the clinical course of CD patients.

Second, Tc17 cells may represent innovative therapeutic targets in IBD. The clear link of these highly pro-inflammatory, polyfunctional T cell responses to disease activity suggests that control of this immune cell population (rather than individual cytokines, such as IL-17 alone), might provide a means to ameliorate disease. This fits to the data on TH17 cells, since “pathogenic” TH17 cells linked to destructive tissue inflammation involved in active disease produce not only IL-17 but also additional pro-inflammatory cytokines and have fundamentally different inflammatory properties not shared by other TH17 subsets^5^. Thus, a strategy for therapeutic approaches should be the control of the disease-associated population rather than targeting the hallmark cytokine IL-17 (which is also produced by non-pathogenic cells). This notion is supported by the failure of a clinical trial using the anti-IL17A monoclonal antibody secukinumab in CD patients^44^. Our identification of a cellular expression signature of this disease-associated subset can facilitate these approaches. This signature includes the molecule CD6. As shown in our study, Tc17, TH17 cells and other proinflammatory T cells can be targeted in vitro using anti-CD6 antibodies. Other approaches targeting Tc17 cells rather than the cytokine could include the selective modulation of type 17 differentiation through modulation of STAT signaling or the use of immunosuppressive reagents that strongly act on Tc17 cells, such as dimethyl fumarate found to induce remission in patients with multiple sclerosis^45^.

The precise mechanisms of anti-CD6 mediated reduction of IL-17 production are unclear. One study had previously reported a significant impact on TH17 differentiation by modulation of STAT3 signaling as a possible mechanism of action of anti-CD6 therapy^36^. Here, even though we observed a reduction of IL-17 production after anti-CD6 intervention, we did not observe a significant modulation of STAT3 signaling in CD8+ T cells. However, CD6 has also been shown to contribute costimulatory signals upon binding ligand CD166/activated leukocyte cell adhesion molecule (ALCAM), enhancing T cell activation via SLP76 phosphorylation^46,47^. Our data show reduced production of pro-inflammatory cytokines upon anti-CD6 treatment, in line with a model of reduced T cell activation. The pronounced reduction in T cell numbers observed in culture, most pronounced for Tc17 cells, further indicate that an effect of anti-CD6 could also be exerted via modulation of proliferation or depletion of target cells. Of note, while we observed a small reduction of cell viability in vitro after Itolizumab addition, this effect did not explain the magnitude of cytokine production changes. Our observation of the strong reduction of CD26^hi^CD161+ T cell frequencies in vitro after 7d culture in the presence of anti-CD6 antibodies fits to previous work describing a role for ALCAM:CD6 interactions in promoting T cell proliferation^48^. Together, our short-term and long-term experimental approaches suggest that use of anti-CD6 antibodies can target Tc17 cells as a population of CD6-expressing T cells effectively and might therefore result in improvement of intestinal inflammation in IBD patients. Anti-CD6 antibodies are in clinical use to treat patients with psoriasis with a reasonable safety profile^37^. However, clinical studies in IBD are currently lacking. Ultimately, prospective clinical trials will be required to define a possible role for anti-CD6 treatment in IBD.

In this work, we identify disease-associated Tc17 cells with a distinct immune expression profile as a key population enriched in active CD that represents an attractive target for therapeutic strategies and as a biomarker for CD patients. A better understanding of this immune cell population is expected to provide opportunities for personalized therapeutic decisions in patients with active disease. Expression of CD6 targetable by monoclonal antibodies in clinical use provides a rationale for this therapeutic strategy in CD patients.

## Methods

### Study approval

Study participants were recruited with approval of the Institutional Review Boards (Ethics committee of the Albert-Ludwigs-University, Freiburg, #407/16 & #14/17, University of Pennsylvania Institutional Review Board #814428). The study was performed in agreement with the principles expressed in the Declaration of Helsinki (2013). Written informed consent was received from participants prior to inclusion in the study.

### Patient populations and specimen collection

CD patients were recruited at the IBD outpatient unit and endoscopy unit of the University Hospital of Freiburg. Additional CD samples were obtained from the biobank of the Immunology in IBD Initiative (I3) at the Hospital of the University of Pennsylvania. Following patients’ informed consent, peripheral blood, intestinal biopsies and clinical parameters including laboratory data were collected. 61 patients with Crohn’s disease and 25 healthy donors were included in the study (Table 1 and Supplementary Table 2–4). For analysis of peripheral blood samples patients were stratified into active and inactive disease based on stool calprotectin concentrations and Harvey Bradshaw Index (HBI)^49^. Intestinal biopsies were stratified into normal and inflamed mucosa based on macroscopic appearance during endoscopy. Due to limited availability of patient sample material and cell numbers per sample not all experiments were performed with all samples, the number of samples is indicated in the respective legend. Each experiment included samples from patients with active disease and in remission. Patient samples were chosen according to these clinical categories as measured by Calprotectin / HBI and based on endoscopic assessment of inflammation and not further selected based on additional parameters.

**Table 1:** CD patients and Healthy donor information.

Patient category	*n*	Age	Sex	HBI	Calprotectin
		(median ± 95% CI)	(% female)	(median ± 95% CI)	(median ± 95% CI)
Healthy donors	25	31 [27; 40]	56%	NA	NA
active CD	33	32 [26; 37]	52%	10 [7; 12]	532 [309; 779]
inactive CD	28	43 [32; 57]	57%	2 [1; 2]	38 [24; 63]

### Lymphocyte isolation

Isolation of mononuclear cells from the peripheral blood and the intestinal tissue was performed as previously described^50,51^. Briefly, isolation of peripheral blood mononuclear cells (PBMC) was done using a ficoll-histopaque density gradient centrifugation (Ficoll Paque PLUS, GE Healthcare Life Sciences). Blood drawn into EDTA tubes was diluted 1:1 with PBS, subsequently overlaid 2:1 on ficoll, and centrifuged for 20 min (800 g, acceleration/ brake 0, room temperature (RT)). The collected buffy coat was washed in phosphate buffered saline (PBS, Corning, #21-040-CV) and pelleted prior to further use (10 min, 500 g, acceleration/brake 9, RT).

Lymphocytes from intestinal biopsies isolated at the University Medical Center Freiburg used for flow cytometric analyses were mechanically homogenized through a 70 µm cell strainer (ThermoFisher, #08-771-2) before washing and biobanking (not subjected to an enzymatic digestion). Samples were obtained during ileocolonoscopy and origin grouped into ileum/colon/sigma. Analysis of biopsies that underwent flow cytometry revealed a mean of 65,449 recorded cells per sample with a minimum of 12,643 and a maximum of 198,257 cells per panel per analysis. For analysis of peripheral blood we plated 1 million PBMCs/well for staining. This amount of cells can in our experience not be reached with endoscopically obtained biopsies. 34.6% of biopsies were from the colon, 34.6% were taken from the ileum and 30.7% from the sigma. Samples were processed and frozen on the day of sample collection and thawed on the day of the respective experiment. For flow cytometry studies this approach was chosen to maximize cell yield and staining performance of chemokine receptors.

For mass cytometry experiments, lamina propria lymphocytes (LPL) and intraepithelial lymphocytes (IEL) were isolated using enzymatic digestion per I3 biobank SOPs. Freeze media (90% FBS (Gem Cell, 100–500), 10% DMSO (Sigma, D2650-5X10ML), DNase I (Sigma, #D5025-150KU, prepared stock of 4 mg/ml) and Collagenase/Dispase (Roche, 500 mg, #11097113001, prepared stock of 50 mg/ml)) were brought to RT. Biopsies were placed into a 50 ml Falcon tube containing 3 ml epithelial strip buffer (1X PBS, 5 mM EDTA (ThermoFisher, #15575020), 1 mM DTT (ThermoFisher, #R0861), 5% FBS, 1% penicillin/streptomycin (Gibco, #15140-122)) and incubated for 10 min in a 37 °C water bath. After vortexing, supernatant was transferred into an Eppendorf tube and spun down (17,000 × *g*, 10 min, 4 °C, accuSpin Micro 17 centrifuge). Supernatant was removed and remaining tissue was further digested in 5 ml of wash buffer (RPMI-1640 (Corning Life Sciences, #10-040-CV), 2% FBS, 1% L-Glutamine (Lonza, #17-605E), 1% pen/strep) supplemented with 50 µl of DNase I and 25 µl of Collagenase/Dispase stocks for 20 min at 37 °C. Following vortexing, the sample was gently strained through a 70 µm strainer and LPL were washed off with 20 ml of wash buffer. Cells were spun down (5 min, 800 × *g*, 4 °C), cryopreserved as described above and thawed on the day of the respective experiment. CyTOF acquisition was performed using LPL samples.

All markers included in the CyTOF panel were previously tested for compatibility with the dissociation protocol and optimized. In order to exclude significant influence of different lymphocyte isolation protocols on the obtained results we validated our findings from the CyTOF experiments in a flow cytometry cohort with a different isolation protocol.

### Cytokine production and multiparametric flow cytometry

For intracellular cytokine staining cells were stimulated with ionomycin (Iono, Sigma, Germany; final concentration 1 µg/ml) and phorbol 12-myristate-13-acetate (PMA; Sigma, Germany; final concentration 50 ng/ml) for 5 h in the presence of brefeldin A (GolgiPlug; BD Biosciences, Germany; 0.5 μL/ml) and monensin (GolgiStop; BD Biosciences, Germany; 0.325 μL/ml) for 4 h at 37 °C. To allow for intracellular staining, cells were treated with the FoxP3 Kit (Thermo Fisher, Germany). 2% paraformaldehyde was used to fix the cells after staining. Flowcytometric analysis was performed with a CytoFLEX (Beckman Coulter, Germany). The following antibodies were used for flow cytometry experiments: CD8 BV650 (RPA-T8, BioLegend), PD-1 BV786 (EH12.1, BD Biosciences), PD-1 BV421 (EH12.2H7, BioLegend), PD-1 PerCP-eFluor710 (eBioJ105, eBioscience), CD26 FITC (2A6, eBioscience), CD6 PE (BL-CD6, BioLegend), CD27 PE-Dazzle594 (M-T271, BioLegend), CD39 PerCP-eFluor710 (eBioA1, eBioscience), CD69 PE-Cy7 (FN50, eBioscience), CD161 APC (191B8, Miltenyi), IFN-γ APC-eFluor780 (4 S.B3, eBioscience), Fixable viability dye BV510 (eBioscience), Fixable viability dye APC-eFluor780 (eBioscience), IL-17 BV605 (BL168, BioLegend), IL-17F BV786 (O33-782, BD Biosciences), RORγt PE (# 600380, R&D Systems), CD56 BV650 (5.1H11, BioLegend), TCRαβ AlexaFluor488 (IP26, BioLegend), TCRαβ BV421 (IP26, BioLegend), TCRγδ PE (5A6.E9, Life Technologies), CD8 PE-Dazzle594 (RPA-T8, BioLegend), TCR Vα7.2 PE-Cy7 (3C10, BioLegend), TCR Vα7.2 FITC (3C10, BioLegend), TCR Vα24Jα18 PE-Cy7 (6B11, Invitrogen), CD3 AlexaFluor700 (SK7, BioLegend), IL-17 PE (eBio64DEC17, eBioscience), TNF PE-Cy7 (MAb11, BioLegend), pSTAT3 Alexa 647 (4/P-STAT3, BD Biosciences), CD3 PerCP (SK7, BD Biosciences), CD8 Krome Orange (B9.11, Beckman Coulter), CD45RA PeCy7 (HI100, BD Biosciences), Isotype IgG2a κ Alexa 647 (MOPC-173, BD Biosciences), CD4 BV786 (L200, BD Biosciences), CD4 BV421 (RPA-T4, BioLegend).

Unstimulated PBMC samples were used to set the gates for flowcytometric analyses and each stimulation experiment included unstimulated PBMC samples. Due to limited cell numbers in biopsies, biopsies were not divided into a stimulated and an unstimulated sample.

### Flow cytometric assessment of conventional and unconventional Tc17 cells

Percentages of γδ T cells, NKT cells and MAIT cells were assessed as fractions of live singlet CD3+ CD8+ CD4- T cells according to published gating strategies^21,52^. Specifically, unconventional T cell populations were identified by subgating for TCR γδ+ (γδ T cells), CD56+ TCR γδ- Vα7.2- (NKT cells), CD161 and Vα7.2+ (CD161^hi^ Vα7.2+ and CD161^mid^ Vα7.2+ MAIT populations) and conventional T cells (TCR γδ- CD56- Vα7.2-) of CD8+ T cells. For TCR γδ assessment, clone 5A6.E9 was used since it strongly stains both Vδ1 and Vδ2 subsets of γδ T cells. In selected experiments, this strategy was validated using a refined gating strategy assessing conventional T cell TCR αβ expression and expression of invariant NKT cell receptor on CD56+ cells using monoclonal 6B11 antibody^34^. Gating strategies are outlined in Supplementary Fig. 5.

### Mass cytometry

Mass cytometry reagents were obtained from Fluidigm or generated by custom conjugation to isotope-loaded polymers using MAXPAR kit (Fluidigm). Mass cytometry antibodies used are shown in Supplementary Table 1. Prior to staining, cells were stimulated with PMA/Ionomycin for 5 h in the presence of golgi inhibitors as described above. Staining was performed as previously described^53^. Briefly, single-cell suspensions were pelleted, incubated with 20 μM Lanthanum-139 (Trace Sciences)-loaded maleimido-mono-amine-DOTA (Macrocyclics) in PBS for 10 min at RT for live/dead discrimination (LD). Cells were washed in staining buffer and resuspended in surface antibody cocktail, incubated for 30 min at RT, washed twice in staining buffer, pre-fixed with PFA 1.6%, washed, then fixed and permeabilized using FoxP3 staining buffer set (eBioscience), and stained intracellularly for 60 min at RT. Cells were further washed twice before fixation in 1.6% PFA (Electron Microscopy Sciences) solution containing 125 nM Iridium overnight at 4 °C. Prior to data acquisition on a CyTOF Helios (Fluidigm), cells were washed twice in PBS and once in dH2O. Mass cytometry data was acquired in one batch using bead-based normalization.

For analysis of mass cytometric data samples were first gated on Iridium intercalator positive, singlet LD negative CD45+ CD3+ CD8+ T cells using FlowJo (v10.6). Further analysis was performed using R (v3.6) (https://www.r-project.org). Analysis was performed using a modified version of the standard workflow of the CATALYST package (version 1.8.7) in Bioconductor^54^. Raw expression values were arcsinh transformed using a cofactor of 5. Clustering was performed using the cluster function from the CATALYST package applied to the following markers: IL-21, IFN-γ, TNF, IL-22, CCL3, IL-2, XCL1, GM-CSF, IL-13, IL-17A, IL-10. Briefly, a FlowSOM clustering was performed with a grid size of 10 × 10 for the self-organizing map followed by metaclustering with ConsensusClusterPlus with a predefined maximum number of 15 clusters. UMAP dimensional reduction was performed using the runDR function from the CATALYST package using the default settings and a random subset of 1000 cells. This package calls the runUMAP function from the scater package which uses the UMAP implementation from the uwot R package. The random seed for all analyses was set to 42. Hierarchical clustering was performed on unscaled data. R script and fcs files are available upon reasonable request.

### Transcriptomic signature analysis

Transcriptional profiling data published by Lee et al.^35^ was extracted from E-MTAB-331 (https://www.ebi.ac.uk/arrayexpress/experiments/E-MTAB-331/), normalized using the vsnrma function from the vsn package, subsetted for common expression features and corrected for batch effects using Combat package in R. Subsequently, hierarchical clustering (hclust) was performed for CD8+ T cells from CD patients using the following markers: CD27, CD6, CD69, DPP4, ENTPD1, KLRB1, PDCD1; patients were stratified into two groups (cutree). Corresponding clinical data was gathered from^35^. Survival analysis was performed using the survminer R package. Patients that reached remission at minimum one timepoint in the follow up were included in the survival analysis (*n* = 31). The R script is available upon reasonable request.

ROC analysis: Time dependent ROC curve for prediction of flare free survival at 200 days of follow up was performed using a GSVA score based on phenotypic Tc17 signature markers CD6, CD69, CD27, CD39, PD1, CD26, CD161 and CD8+ T cell transcriptome data and corresponding clinical data from 35 untreated CD patients that were extracted from E-MTAB-331^35^.

### T cell expansion under IL-17 polarizing conditions

For cultivation under IL-17 polarizing conditions cells were plated at 2 million PBMC/well in a sterile flat-bottom 96 well plate in complete medium (RPMI 1640 with 10% fetal calf serum, 1% penicillin/streptomycin solution and 1.5% 1 M HEPES; all Thermo Fisher, Germany) and stimulated with 25 µl/ml T cell activator (Stemcell Technologies, Canada). Cytokines were added to achieve the following final concentrations: 12.5 ng/ml for IL-1β, 5 ng/ml for TGFβ, 25 ng/ml for IL-6 (all Stemcell Technologies, Canada) and 25 ng/ml for IL-23 (Miltenyi Biotec, Germany). Cells in the control condition were stimulated with T cell activator only. Cells were cultured for 7 days at 37 °C, counted on days 3, 5, 7 and analyzed by flow cytometry on day 3 and 7 after restimulation with PMA/ionomycin.

### STAT3 phosphorylation experiments

2 million PBMC/well were stimulated with IL-21 (10 ng/ml) (Miltenyi Biotec, Germany) in IMDM for 15 min at 37 °C with or without prior addition of Itolizumab (40 µg/ml) overnight and/or 15 min prior to stimulation. Cells were then treated with the BD Phosflow kit (BD Biosciences, Germany) according to manufacturer’s instructions and STAT3 phosphorylation was assessed via flow cytometry.

### Anti-CD6 antibodies

Itolizumab (Alzumab™, clone CD6D1, Biocon, India) was added to lymphocyte cell cultures at a final concentration of 40 µg/ml and anti-CD6 antibody, clone UMCD6 (Sigma–Aldrich, Germany) was added to a final concentration of 10 µg/ml for the respective experiments. Isotype antibodies were used to control for the addition of blocking antibodies. The following isotype control antibodies were used: For Itolizumab: purified human IgG1, κ isotype control recombinant antibody (clone QA16A12, BioLegend, USA) at a final concentration of 40 µg/ml, for UMCD6: purified mouse IgG1, κ isotype control antibody (clone MG1-45, BioLegend, USA) at a final concentration of 10 µg/ml.

### Statistics

FlowJo software v10.6 (FlowJo LLC, USA) was used to analyze flow cytometric and mass cytometric data. Statistical analysis was performed using GraphPad version 8 (Prism Software Inc., USA) and R version 3.6 (https://www.r-project.org) respectively. Essential R packages used are: SummarizedExperiment, CATALYST, flowCore, ComplexHeatmap, limma, lme4, edgeR, FlowSOM, tsne, nlme, MASS, Rtsne. Statistical tests used: Kruskal-Wallis-Test with Dunn’s multiple comparison’s test (Figs. 1A, C, D, F, 4), Mann–Whitney test (Figs. 1F, 5C), Wilcoxon test (Figs. 3,  6D–F), Log rank test (Fig. 5A). Marginal means in a linear model were used to assess statistical significance in Fig. 6C. A *p* value < 0.05 was considered significant. If not specified otherwise, ****indicates a *p* value <0.0001, *** <0.001, ** <0.01, * <0.05. Flow cytometric and mass cytometric original data plots are depicted using FlowJo as 2% contour plot with outlier setting or dot plots.

### Reporting summary

Further information on research design is available in the Nature Research Reporting Summary linked to this article.

## Supplementary information

Supplementary Information

Reporting Summary

## Data Availability

Data are present in the manuscript and supplementary material and source cytometry files and R scripts generated during the current study are available from the corresponding author upon request.
