# Supplementary material for: High-dimensional profiling reveals Tc17 cell enrichment in active Crohn’s disease and identifies a potentially targetable signature
Source: Nat Commun. 2022 Jun 27;13:3688. doi: 10.1038/s41467-022-31229-z (PMC9237103; doi:10.1038/s41467-022-31229-z)
Supplement: Supplementary file 1 — Supplementary Information [file 41467_2022_31229_MOESM1_ESM.pdf]

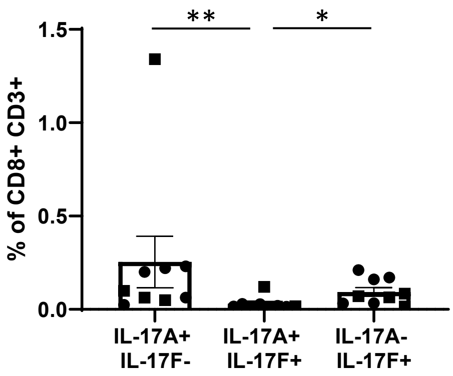

Supplementary Figure 1 (related to Main Figure 1) | IL-17A and IL-17F production by CD8+ CD3+ cells.

IL-17A and IL-17F coproduction is quantified as a fraction of CD8+ T cells. HD (dots, n=5) and CD patients (squares, n=4). Kruskal-Wallis test was used to assess statistical significance. Dunn's test was used to adjust for multiple comparisons. IL-17A+IL-17F- vs IL-17A+ IL-17F+: p <0.01, IL-17A+IL-17F+ vs IL-17A-IL-17F+: p <0.05. Data are presented as mean +/- SEM. Statistical tests used were two-sided.

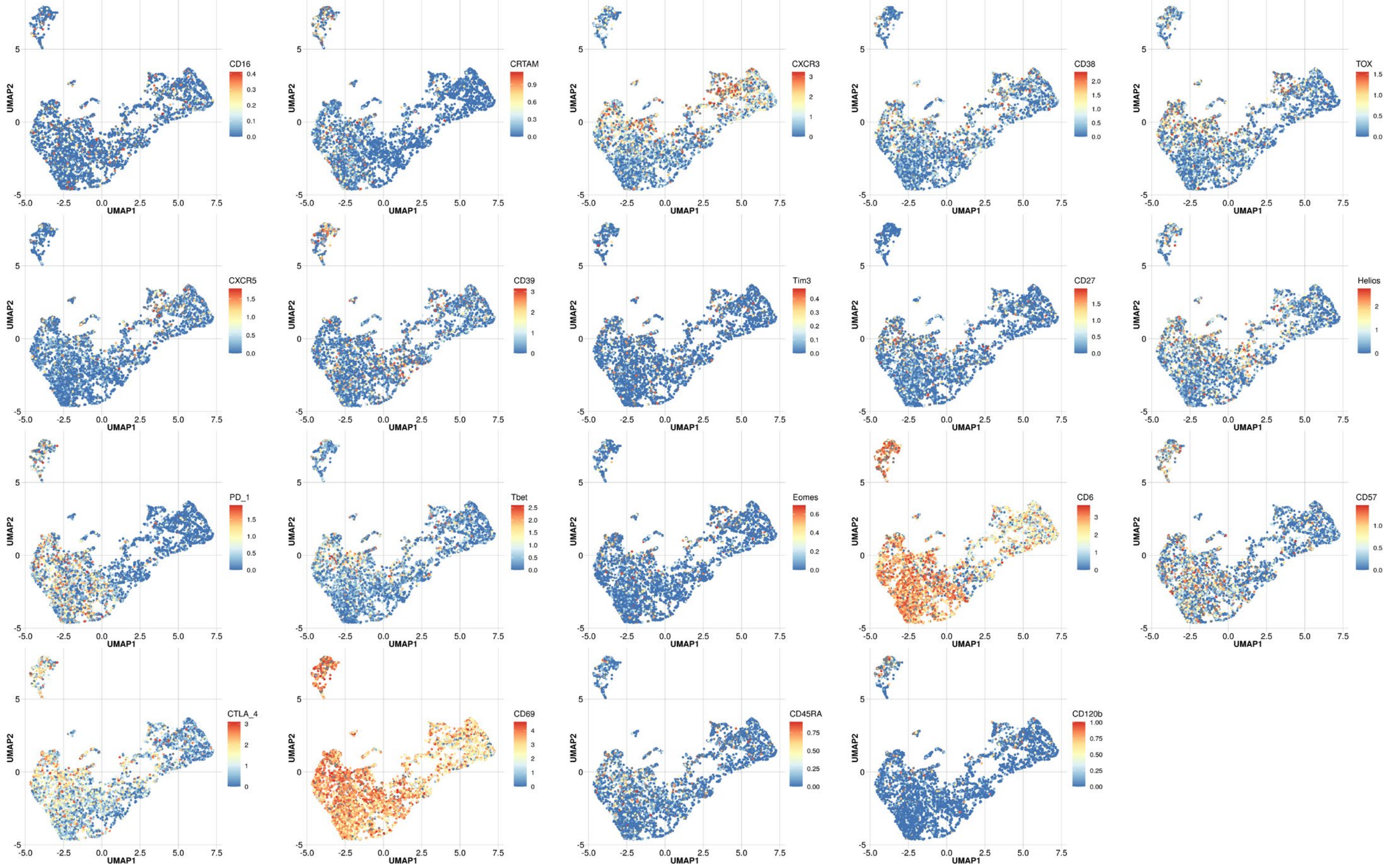

B

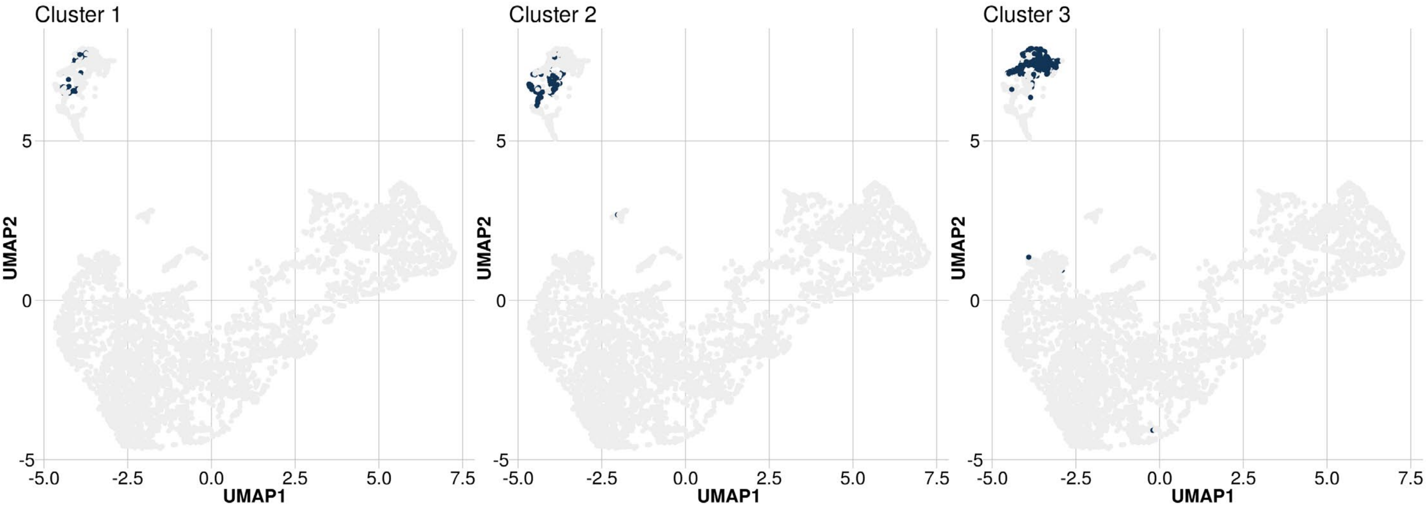

Supplementary Figure 2 (related to Main Figure 2) | High dimensional mass cytometry analysis reveals a novel signature of IL-17 producing CD8 T cells.

UMAP analysis was performed on mass cytometry data obtained from intestinal tissue samples of 3 CD patients. A) Live singlet CD45+CD3+CD8+ T cells are shown on the UMAP, with expression of immune markers depicted in Fig. 2D plotted with heatmap coloring. Color scale is indicated adjacent to each plot and was calculated from 0 to 95th quantile of arcsinh transformed MSI. B) Tc17 clusters 1, 2 and 3 identified by FlowSOM metaclustering as in Fig. 2B-F are individually plotted as individual black dots on the UMAP, with all other CD8 T cells displayed in light grey.

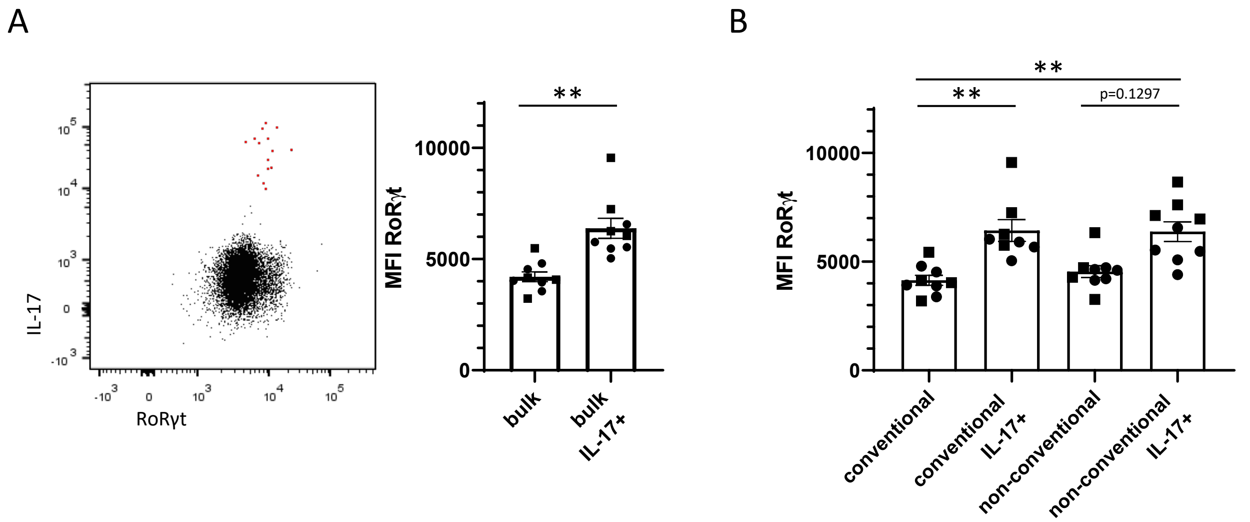

Supplementary Figure 3 (related to Main Figure 2 and 4) | Expression of the transcription factor RoRγt by IL-17 producing cells in the peripheral blood.

A) Representative FACS plot depicting RoRγt expression by IL-17+ CD8+ CD3+ live lymphocytes (red) versus IL-17- CD8+ CD3+ live lymphocytes (black). Dots represent healthy donors (n=5), squares represent CD patients (n=4). Wilcoxon test was used to assess statistical significance. Data are presented as mean +/- SEM. Statistical tests used were two-sided, $p < 0.01$ B) MFI of RoRγt on different CD8+ subpopulations; conventional CD8+ cells were identified as Vα7.2-, Vα24Jα18- and αβTCR+, non-conventional CD8 T cells by boolean exclusion. Dots represent healthy donors (n=5), squares represent CD patients (n=4). Kruskal-Wallis test was used to assess statistical significance and Dunn's test was used to adjust for multiple testing. Data are presented as mean +/- SEM. Statistical tests used were two-sided; conventional vs conventional IL-17+ $p < 0.01$; conventional vs non-conventional IL-17+ $p < 0.01$.

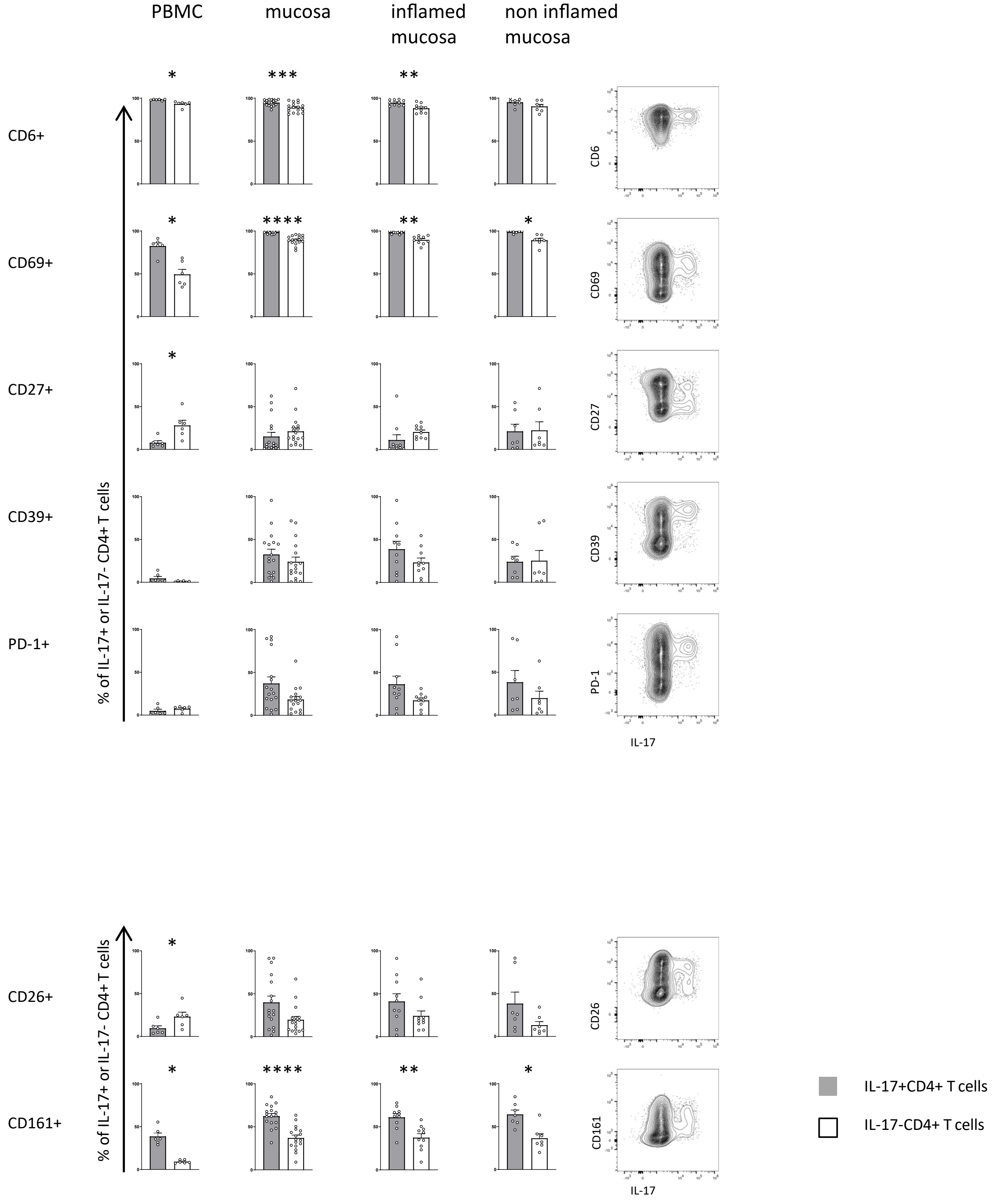

Supplementary Figure 4 (related to Main Figure 3) | Analysis of the novel Tc17 signature in CD4 T cells.

The Tc17 signature markers were analyzed between IL-17 producing (grey) and non-IL-17 producing CD4 T cells (white) from the peripheral blood (n=6), the mucosa (n=17), the inflamed mucosa (n=10) and the non-inflamed mucosa (n=7). Representative FACS plots depict Tc17 signature markers gated against IL-17 production by CD4 T cells isolated from an inflamed biopsy from the sigma of a CD patient. Wilcoxon test was used to assess statistical significance. Data are presented as mean +/- SEM. Statistical tests used were two-sided.
**** indicates a p value <0.0001, *** <0.001, ** <0.01, * <0.05.

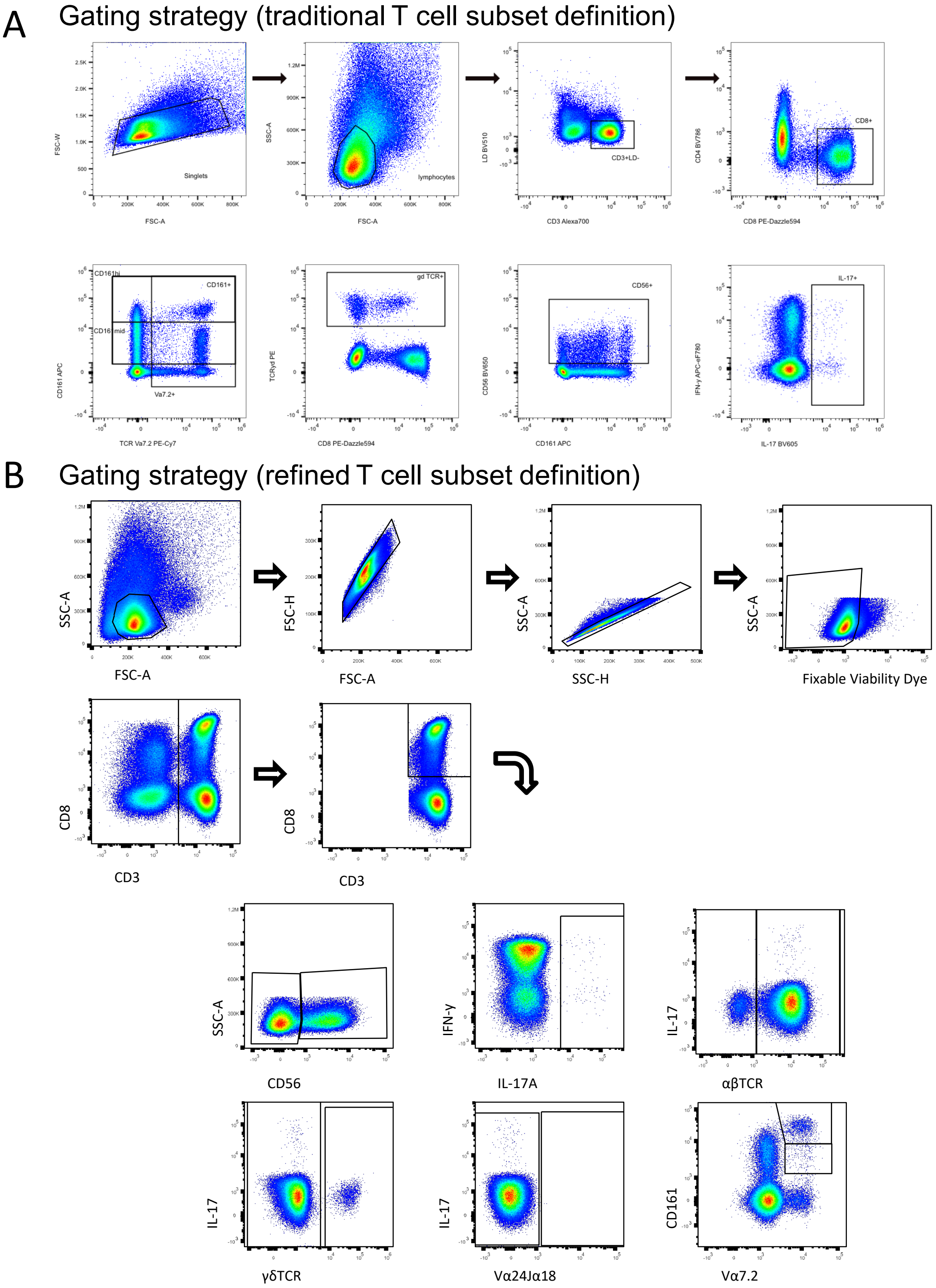

Supplementary Figure 5 (related to Main Figure 4) | Gating strategy for T cell subset identification.

A) Traditional T cell subset definition. The percentage of γδ T cells (TCR γδ+ non-NKT, non-MAIT), NKT cells (CD56+ TCR γδ- Vα7.2-), MAIT cells (CD161hi Vα7.2+ or CD161mid Vα7.2+) and conventional T cells (TCR γδ- CD56- Vα7.2-) of all CD8 T cells were determined by gating and boolean gate definitions. All gates were defined on CD3+ cells and consecutively applied to CD8+CD3+ and IL-17+CD8+CD3+ cells. Boolean gate definitions: (traditional): γδ T cells: γδ TCR+ & non-MAIT, non-NKT; MAIT hi: CD161hi+ & Vα7.2+; MAIT mid: CD161mid+ & Vα7.2+; CD56+ NKT cells: CD56+ & Vα7.2- & TCRγδ- B) Refined gating strategy used for identification of T cell subpopulations including staining for invariant Vα24Jα18 TCR (clone 6B11 antibody). Boolean gate definitions: (refined): γδ T cells: γδ TCR+ & αβ TCR-; MAIT hi: αβ TCR+ & CD161hi+ & Vα7.2+; MAIT mid: αβ TCR+ & CD161mid+ & Vα7.2+; CD56+ NKT cells: CD56+ & αβ TCR+ & Vα7.2- & TCRγδ-

intestine

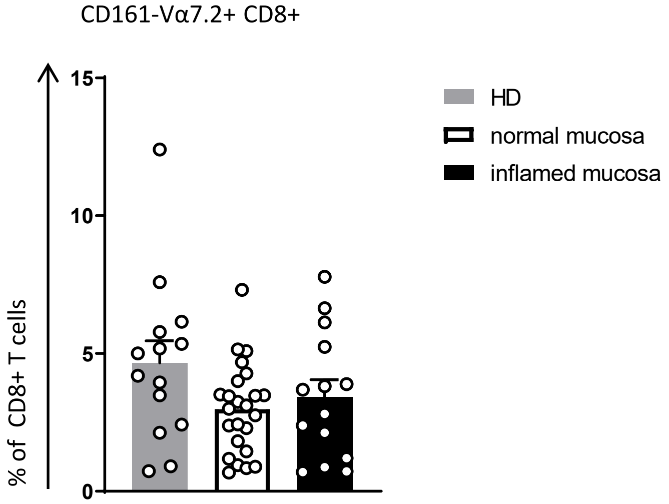

Supplementary Figure 6 (related to Main Figure 4) | Frequency of CD161-Vα7.2+CD8 T cells in the intestinal mucosa.

Frequency of CD161-Vα7.2+CD8 T cells in intestinal mucosa of healthy donors (n biopsies= 14), in non inflamed intestinal mucosa of CD patients (n biopsies= 24) and inflamed intestinal mucosa of CD patients (n biopsies= 14) was assessed. Data are presented as mean +/- SEM.

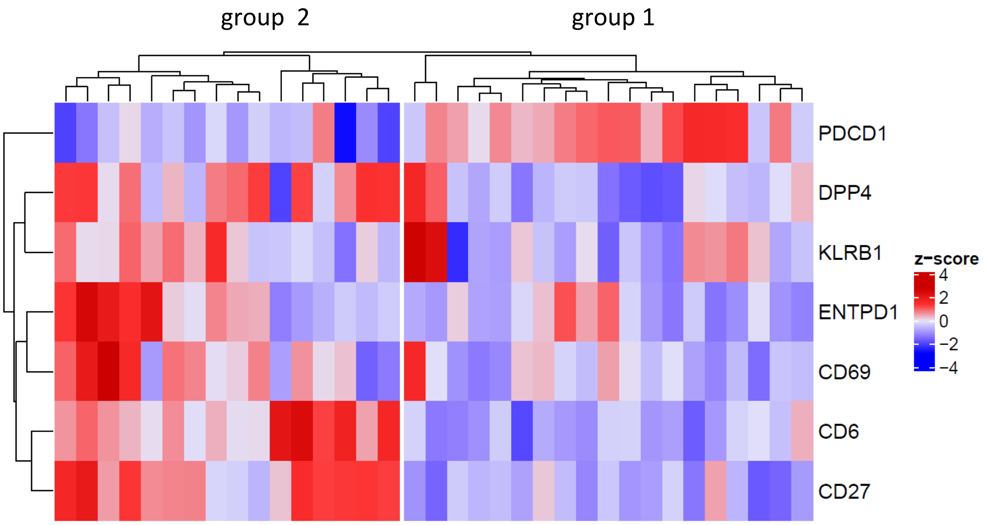

Supplementary Figure 7 (related to Main Figure 5) | Heatmap of gene expression of signature markers of the novel Tc17 signature.

Hierarchical clustering was performed on batch corrected microarray expression values (E-MTAB-331) based on PDCD1, DPP4, KLRB1, ENTPD1, CD69, CD6 and CD27. Expression is depicted by z-score in the heatmap. Hierarchical clustering revealed two distinct populations as shown in Fig. 5A, B.

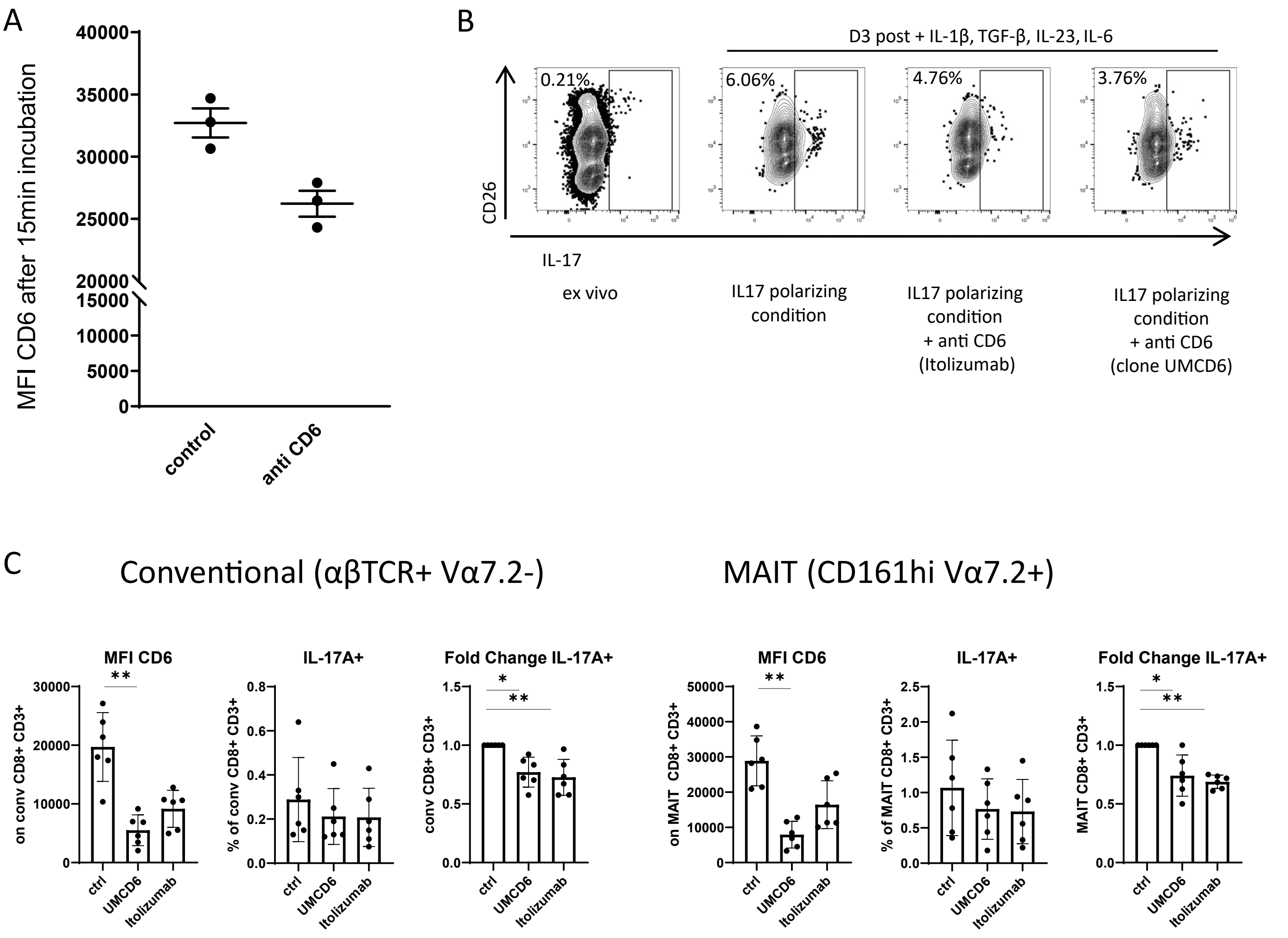

Supplementary Figure 8 (related to Main Figure 6) | Targeting CD6 expressed by different conventional and unconventional T cells reduces IL-17 production.

A) Incubation with anti-CD6 antibody rapidly leads to a decrease in staining MFI of CD6 on CD8 T cells. The MFI of CD6 on CD8 T cells is shown after incubation of PBMC with anti-CD6 antibody (Itolizumab) for 15 minutes prior to staining, n=3 individual donors. Data are presented as mean +/- SEM. B) Decreased IL-17 production in presence of anti-CD6. Frequency of Tc17 cells ex vivo and after 3 days of culture under IL-17 polarizing conditions +/- anti-CD6 antibody (Itolizumab vs clone UMCD6) is shown on representative FACS plots gated on CD8 T cells. C) Expression of CD6 and production of IL-17 by CD8 T cells after stimulation with PMA and ionomycin for 5 h in control conditions and in the presence of antibodies targeting CD6 (UMCD6 or Itolizumab) (n=6) was assessed for CD161hiVα7.2+ CD8+ MAIT cells and conventional (αβTCR+ Vα7.2-) CD8 T cells. Kruskal-Wallis test was used to assess statistical significance and Dunn's test was used to adjust for multiple comparisons. Data are presented as mean +/- SD. Statistical tests used were two-sided. Conventional: MFI CD6: ctrl vs UMCD6 $p < 0.01$; Fold change IL-17A+: ctrl vs UMCD6 $p < 0.05$, ctrl vs Itolizumab $p < 0.01$. MAIT: MFI CD6: ctrl vs UMCD6 $p < 0.01$; Fold change IL-17A+: ctrl vs UMCD6 $p < 0.05$, ctrl vs Itolizumab $p < 0.01$.

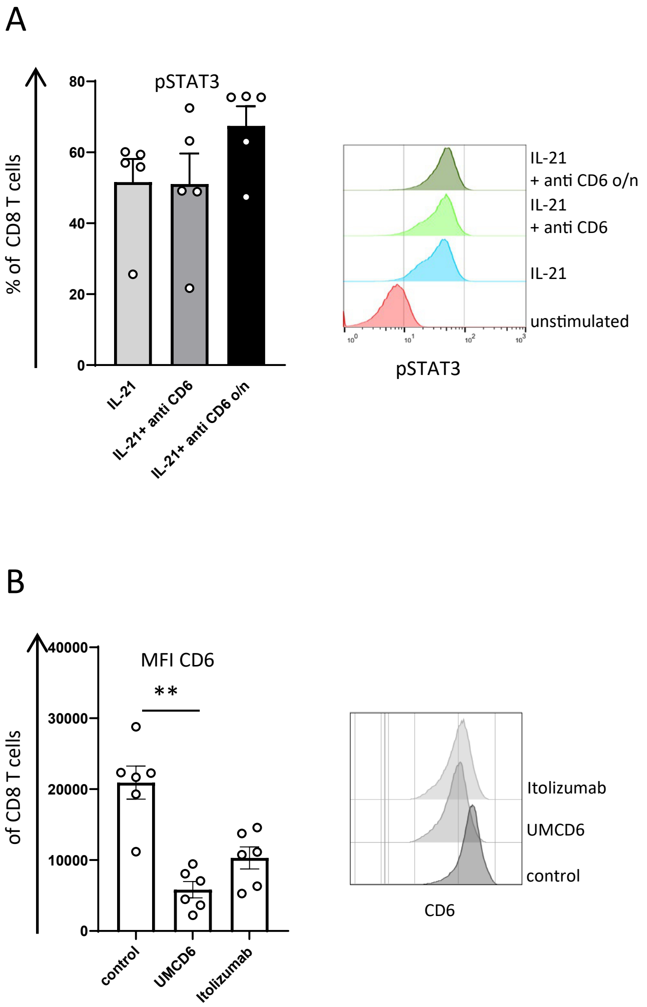

Supplementary Figure 9 (related to Main Figure 6) | Targeting of CD6 *in vitro* does not modulate STAT3 signaling in CD8 T cells.

A) STAT3 phosphorylation was assessed in CD8 T cells after PBMC stimulation with IL-21 for 15min +/- anti-CD6 (Itolizumab) present during stimulation or overnight pre-incubation with anti-CD6 (n=5). Data are presented as mean +/- SEM. B) Expression of CD6 by CD8 T cells from the peripheral blood of healthy donors (n=6) after stimulation with PMA and ionomycin for 5 h in control conditions and in the presence of antibodies targeting CD6 (UMCD6 or Itolizumab). Kruskal-Wallis test was used for statistical testing and Dunn's test to adjust for multiple testing. Data are presented as mean +/- SEM. Statistical tests used were two-sided, $p < 0.01$.

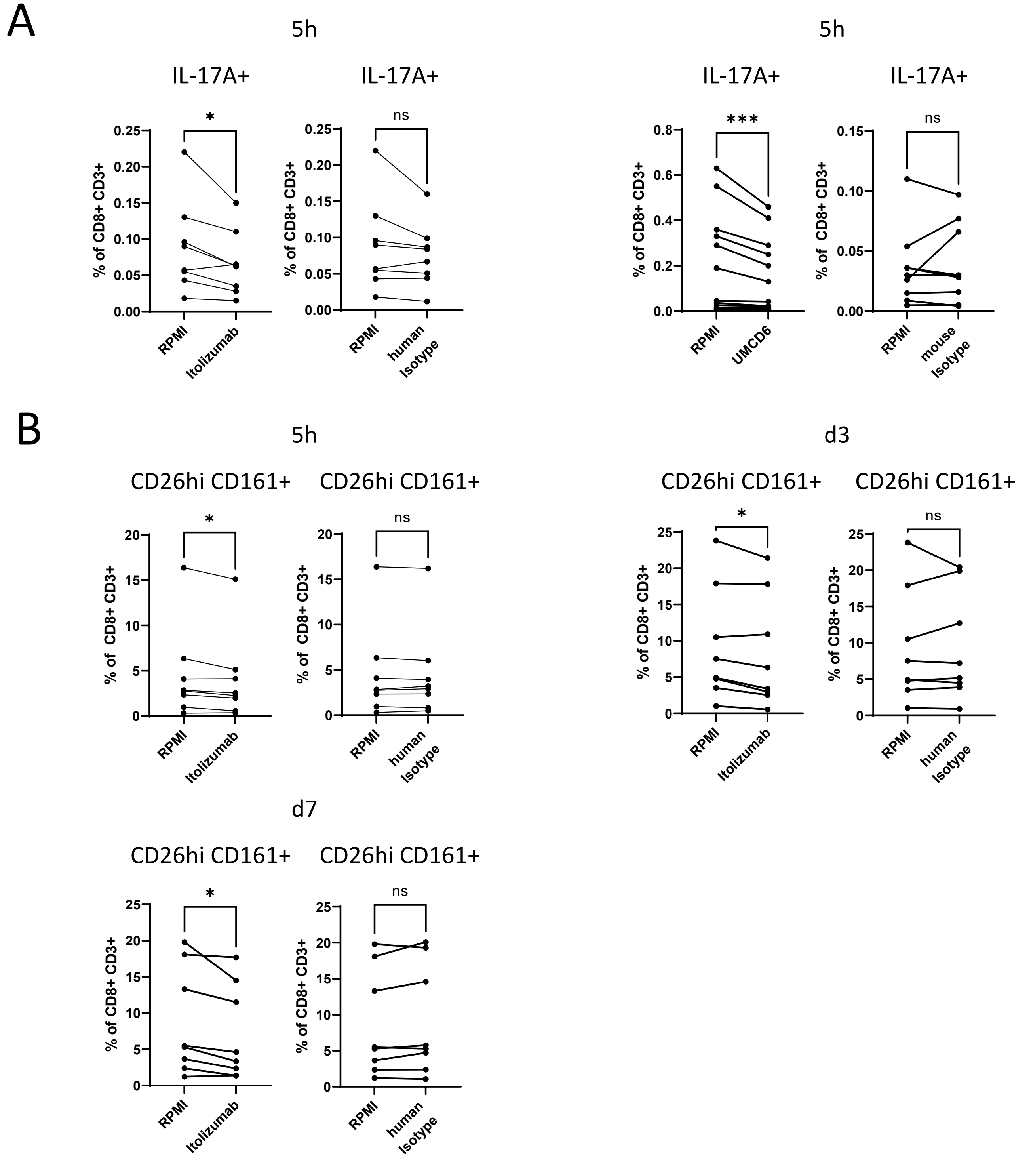

Supplementary Figure 10 (related to Main Figure 6) | Anti-CD6 reduces proinflammatory Tc17 cells.

A) Percentage of IL-17A+ cells of CD8+ T cells after 5h stimulation with PMA/Ionomycin in presence of anti-CD6 antibodies Itolizumab or UMCD6 or equal amounts of respective sotype controls – human isotype antibody for Itolizumab and mouse isotype antibody for UMCD6. Statistical tests used were two-sided. RPMI vs Itolizumab $p < 0.05$; RPMI vs MCD6 $p < 0.001$.
B) Percentage of CD26hi CD161+ cells of CD8+ T cells after 5h stimulation with PMA/ Ionomycin or three or seven-day culture in presence of anti-CD6 antibodies or respective Isotype controls. Wilcoxon test was used to determine statistical significance. Statistical tests used were two-sided. RPMI vs Itolizumab $p < 0.05$ for 5h, d3 and d7.

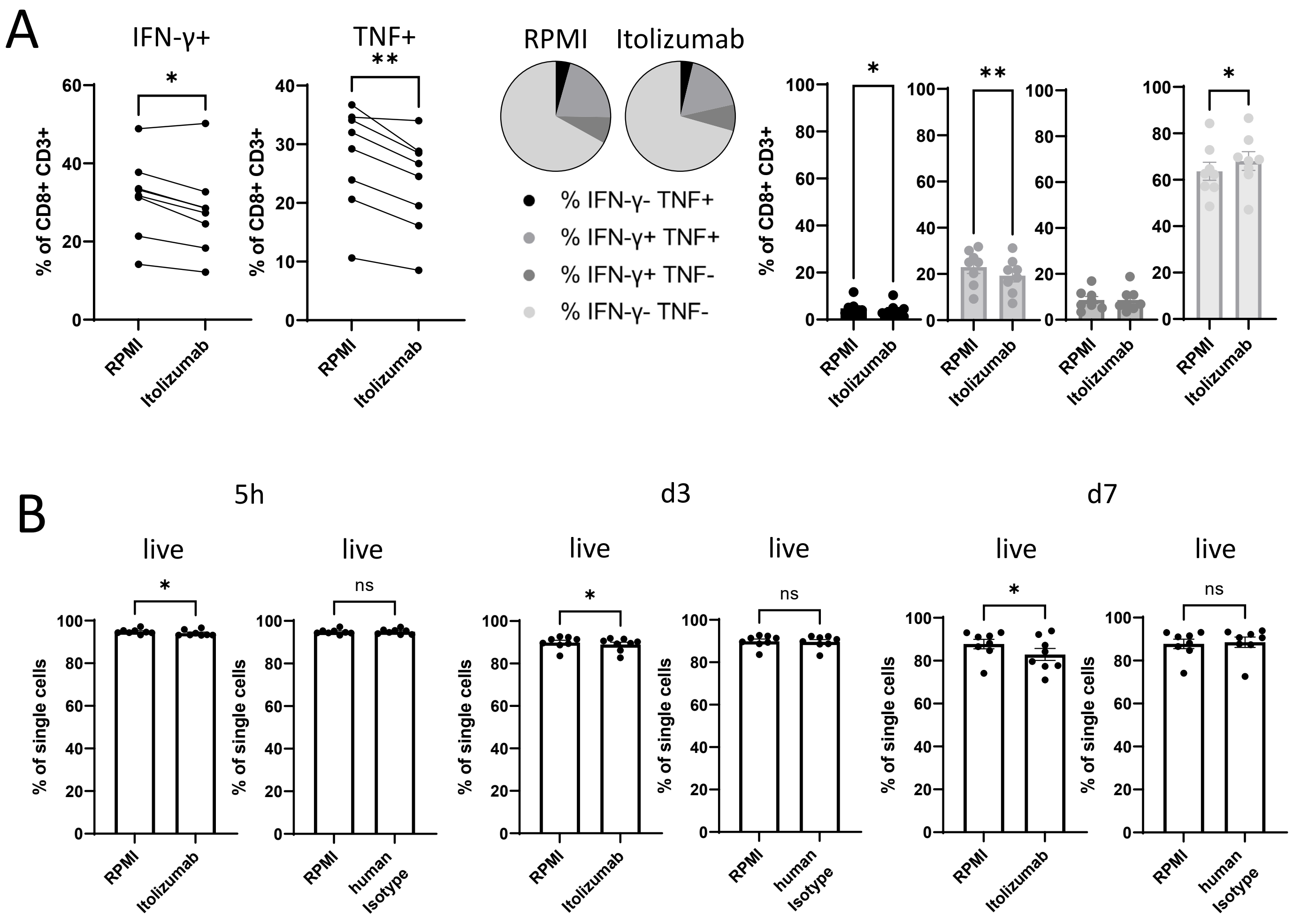

Supplementary Figure 11 (related to Main Figure 6) | Polyfunctionality of CD8+ cells after anti-CD6 blockade.

A) Analysis of IFN-γ and TNF producing cells of CD8+ T cells after stimulation with PMA/Ionomycin for five hours in presence of indicated antibodies (n = 8). Data are presented as mean +/- SEM. Statistical tests used were two-sided. Left panel: IFN- γ $p < 0.05$, TNF $p < 0.01$. Right panel: IFN-γ-TNF+ $p < 0.05$; IFN-γ+ TNF+ $p < 0.01$; IFN-γ-TNF- $p < 0.05$. Pie chart representing median percentages. B) Percentage of live cells of single cells assessed by flow cytometric staining after stimulation with PMA/Ionomycin, three days or seven days of culture in presence of the indicated antibodies (n = 8). Data are presented as mean +/- SEM. Statistical tests used were two-sided. $P < 0.05$ for RPMI vs Itolizumab on 5h, d3 and d7 timepoint. For statistical testing, Wilcoxon match-paired test was used.

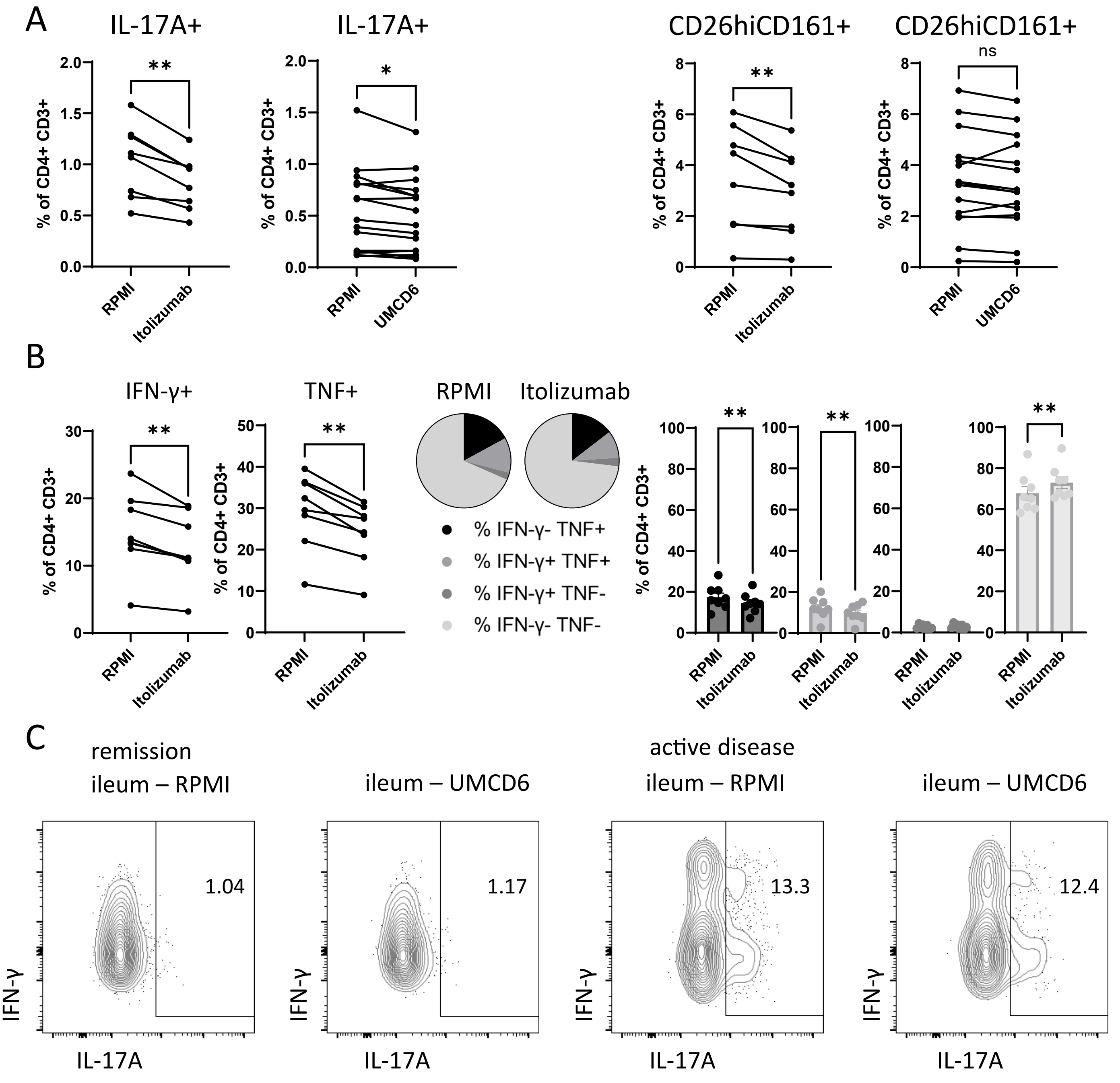

Supplementary Figure 12 (related to Main Figure 6) | Anti-CD6 limits the proinflammatory cytokine profile of TH17 cells.

A) percentage of IL-17A positive cells of CD4+ T cells from the peripheral blood of healthy donors after five-hour-stimulation with PMA/Ionomycin in presence of anti-CD6 antibodies Itolizumab (n = 8) or UMCD6 (n = 14), compared to RPMI. Percentage of CD26hiCD161 positive cells of CD4+ T cells after stimulation in presence of anti-CD6 antibodies Itolizumab (n = 8) or UMCD6 (n= 14), compared to RPMI. Wilcoxon test was used to determine statistical significance. Statistical tests used were two-sided. IL-17A+ RPMI vs Itolizumab: $p < 0.01$; RPMI vs UMCD6: $p < 0.05$; CD26hiCD161+ RPMI vs Itolizumab $p < 0.01$. B) Analysis of IFN-γ and TNF producing cells of CD4+ T cells after stimulation with PMA/Ionomycin for five hours in presence of indicated antibodies (n = 8). Pie chart representing median percentages. Data are presented as mean +/- SEM. Statistical tests used were two-sided. Left panel: IFN-γ+: $p<0.01$, TNF+: $p<0.01$. Right panel: IFN-γ-TNF+ $p <0.01$; IFN-γ+TNF+ $p <0.01$; IFN-γ-TNF- $p <0.01$. C) Representative plots of IL-17A+ CD4+ T cells from ileal biopsies of patients suffering from Crohn's disease. Cells were stimulated with PMA/ Ionomycin for five hours in presence of the indicated antibodies. For statistical testing, Wilcoxon test was performed.

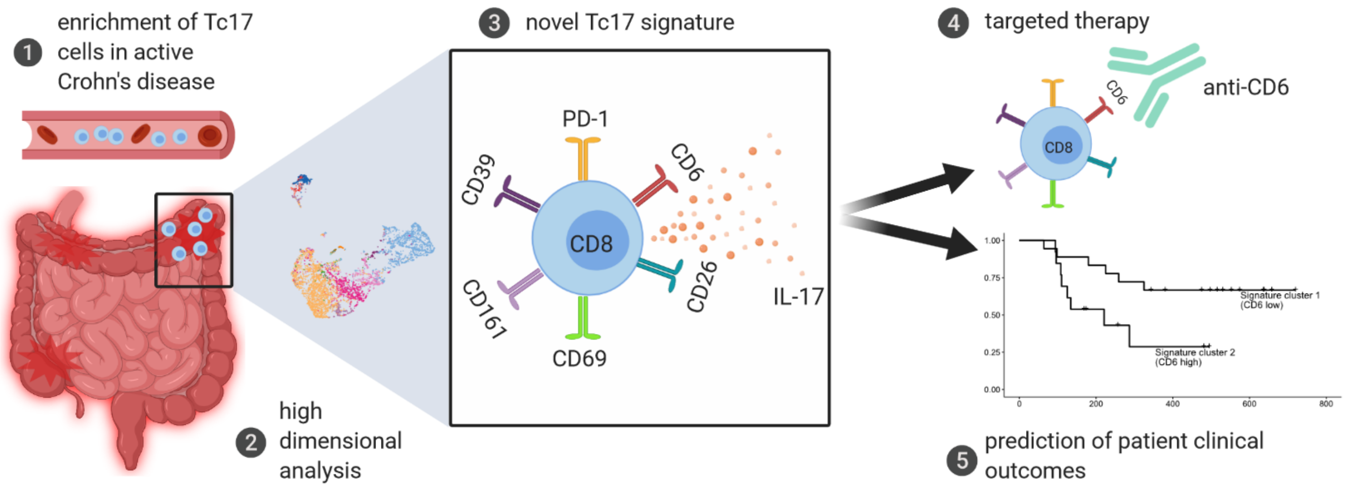

Supplementary Figure 13 | Graphical abstract summarizing key findings of this work. This figure was created with BioRender.com.

Suppl. Table 1: Mass cytometry antibodies.

Channel	Marker	Clone	Source	Catalog#
**89 Y**	CD45	HI30	Fluidigm	3089003B
**113 In**	CD6	MT605	BD	555356
**115 In**	CD57	TB01	Ebioscience	MA5-16948
**141 Pr**	CD3	UCHT1	Biolegend	300402
**142 Nd**	IFN-G	B27	Biolegend	506501
**143 Nd**	CD4	RPA-T4	Biolegend	300502
**144 Nd**	CTLA-4	BNI3	BD	555851
**145Nd**	TNF	MAb11	Ebioscience	14-7349-81
**146 Nd**	CD8	RPA-T8	Biolegend	301002
**147 Sm**	CD45RA	H100	BD	555486
**148 Nd**	CD69	FN50	Biolegend	310902
**149 Sm**	CCL3	MAB2701	R&D	MAB2701-100
**150 Nd**	IL-22	22URTI	Fluidigm	3150007B
**151 Eu**	CD39	A1	Biolegend	328221
**152 Sm**	IL-2	MQ1-17H12	Ebioscience	14-7029-81
**153 Eu**	TIM-3	F38-2E2	Fluidigm	3153008B
**154 Sm**	XCL-1	109001	R&D	MAB6951
**155 Gd**	CD27	L128	Fluidigm	3155001B
**156 Gd**	Helios	22F6	Biolegend	137202
**158 Gd**	PD-1	EH12.2H7	Biolegend	329902
**159 Tb**	GM-CSF	BVD2-21C11	Fluidigm	3159008B
**160 Gd**	Tbet	4B10	Fluidigm	3160010B
**161 Dy**	CRTAM	Cr24.1	Biolegend	339102
**162 Dy**	IL-21	3A3-N2	Biolegend	513001
**163 Dy**	CXCR3	G025H7	Biolegend	353702
**164 Dy**	LIFR	1C7	EMD	MABD150
**165 Ho**	Eomes	WD1928	Ebioscience	14-4877-82
**166 Er**	Areg	polyclonal	Thermo	RB-257-PABX
**167 Er**	CD38	HIT2	Fluidigm	3167001B
**168 Er**	Tox	Rea473	Miltenyi	custom
**169 Tm**	Il-13	JES10-5A2	Fluidigm	3169016B
**170 Er**	CXCR5	RF8B2	BD	552032
**171 Yb**	CADM1	polyclonal	EMD	ABT66
**172 Yb**	IL-17A	BL168	Fluidigm	3172020B
**173 Yb**	CD120B	hTNFR-M1	Biolegend	551311
**174 Yb**	IL-10	JES3-12GB	Biolegend	501505
**175 Lu**	OSM	17022	R&D	MAB2951
**176 Yb**	LIF	M1506B09	Biolegend	674702
**209 Bi**	CD16	3G8	Fluidigm	3209002B

Suppl. Table 2: Healthy donors.

Patient	Age	Gender
**HD-1**	28	m
**HD-2**	55	f
**HD-3**	63	m
**HD-4**	29	m
**HD-5**	48	f
**HD-6**	27	f
**HD-7**	58	f
**HD-8**	27	f
**HD-9**	26	f
**HD-10**	23	f
**HD-11**	24	m
**HD-12**	29	m
**HD-13**	38	m
**HD-14**	40	m
**HD-15**	44	f
**HD-16**	38	m
**HD-17**	48	f
**HD-18**	31	f
**HD-19**	44	m
**HD-20**	32	m
**HD-21**	22	f
**HD-22**	24	f
**HD-23**	22	f
**HD-24**	23	m
**HD-25**	31	f

Suppl. Table 3: Patients with active Crohn's disease.

Patient	Calprotectin (mg/kg)	HBI	Age	Gender	Mesalazine	Budesonide	Prednisone	Thiopurines	Methotrexate	Anti-TNF antibodies	Anti-Integrin antibodies	Other therapies	Montreal Classification
**Infl-1**	655	13	29	f	previously	previously	previously	previously	never	currently	never	Anti IL-23	A2L4+B3p
**Infl-2**	413	10	37	m	previously	previously	previously	never	never	previously	never	none	A2L4+B3p
**Infl-3**	512	7	37	f	never	never	currently	currently	never	never	never	none	A2L2B1
**Infl-4**	NA	12	26	f	never	previously	previously	previously	never	currently	never	none	A2L4+B3p
**Infl-5**	414	9	48	m	previously	previously	previously	previously	never	previously	never	none	A2L4+B3p
**Infl-6**	309	14	26	f	never	never	previously	previously	never	never	never	none	A2L4+B3p
**Infl-7**	635	11	29	m	previously	previously	previously	never	never	currently	never	none	A2L3B1
**Infl-8**	NA	1	32	m	previously	never	never	never	never	never	never	none	A2L3B3p
**Infl-9**	822	>6	30	f	previously	previously	previously	previously	never	previously	never	none	A2L2B2
**Infl-10**	NA	9	42	m	previously	previously	previously	previously	never	previously	never	none	A2L3B3p
**Infl-11**	1555	7	42	m	previously	currently	previously	previously	never	currently	never	none	A2L3B3p
**Infl-12**	NA	16	47	f	previously	previously	previously	previously	never	currently	never	none	A2L3B2
**Infl-13**	532	6	36	m	previously	never	previously	never	previously	currently	never	none	A2L3B2
**Infl-14**	2000	11	32	f	previously	previously	previously	previously	previously	currently	never	none	A1L4+B3p
**Infl-15**	969	9	34	f	previously	previously	previously	previously	never	currently	never	none	A2L3B1
**Infl-16**	300	7	35	m	never	never	previously	previously	previously	currently	never	none	A1L4+B3p
**Infl-17**	720	12	18	m	never	never	previously	currently	never	currently	never	none	A1L4B1
**Infl-18**	752	10	31	f	previously	previously	previously	previously	never	currently	never	none	A1L4+B3p
**Infl-19**	82	20	50	f	previously	previously	previously	currently	never	previously	never	Immunoglobulins	A2L2B2
**Infl-20**	485	13	38	f	previously	previously	previously	previously	never	never	never	none	A2L4+B3p
**Infl-21**	779	3	30	m	previously	previously	currently	previously	never	previously	currently	cyclosporine	A1L3B1
**Infl-22**	1067	1	20	m	previously	previously	previously	previously	never	currently	never	none	A1L3B1
**Infl-23**	181	0	23	m	never	previously	previously	currently	never	never	never	none	A1L3B3
**Infl-24**	114	5	55	m	previously	previously	previously	never	never	currently	never	none	A2L3B3p
**Infl-25**	NA	6	19	f	never	previously	previously	previously	never	currently	never	none	A1L3B1
**Infl-26**	155	3	54	f	previously	previously	previously	previously	never	currently	never	none	A3L1B2
**Infl-27**	NA	NA	21	m	previously	never	previously	never	previously	currently	never	none	NA
**Infl-28**	NA	23	20	f	previously	previously	previously	previously	previously	previously	previously	none	A1L3B2
**Infl-29**	NA	12	61	m	previously	previously	currently	currently	previously	previously	never	none	A2L1B3
**Infl-30**	NA	20	21	f	previously	previously	previously	never	never	previously	never	Ustekinumab	A2L4+B3p
**Infl-31**	NA	17	18	f	never	never	currently	never	never	currently	never	none	A2L3B2
**Infl-32**	NA	26	18	m	previously	previously	previously	previously	previously	previously	never	Ustekinumab	A1L4B2
**Infl-33**	NA	10	32	f	previously	previously	previously	currently	never	currently	previously	Ustekinumab (previously)	A2L2B3p

Suppl. Table 4: Patients with inactive Crohn's disease

Patient	Calprotectin (mg/kg)	HBI	Age	Gender	Mesalazine	Budesonide	Prednisone	Thiopurines	Methotrexate	Anti-TNF antibodies	Anti-Integrin antibodies	Other therapies	Montreal Classification
**Non Infl-1**	45	1	31	f	previously	previously	previously	previously	never	currently	never	none	A2L3B3p
**Non Infl-2**	80	3	49	m	previously	previously	previously	previously	never	currently	never	none	A2L2B1
**Non Infl-3**	27	1	75	f	never	never	never	never	never	never	never	none	A3L4+B3p
**Non Infl-4**	37	2	70	m	never	previously	never	never	never	never	never	none	A3L3B2
**Non Infl-5**	63	4	31	f	currently	previously	previously	never	never	currently	never	none	A2L3B1
**Non Infl-6**	71	2	70	f	previously	previously	previously	previously	never	never	never	none	A3L4B1
**Non Infl-7**	24	5	51	f	never	never	previously	never	previously	currently	never	none	A3L1B1
**Non Infl-8**	39	2	58	m	previously	previously	previously	previously	never	currently	never	none	A2L3B2
**Non Infl-9**	77	1	77	f	never	never	previously	never	never	never	never	sulfasalazine	A3L33B3
**Non Infl-10**	32	4	45	m	previously	previously	previously	previously	never	currently	never	none	A2L3B3p
**Non Infl-11**	25	3	37	m	never	previously	previously	currently	never	never	never	none	A2L1B1
**Non Infl-12**	55	2	63	f	never	previously	previously	currently	never	currently	never	none	A2L1B3
**Non Infl-13**	59	2	66	m	previously	currently	previously	never	never	never	never	none	A2L1B3
**Non Infl-14**	26	0	57	m	currently	never	previously	never	never	never	never	sulfasalazine	A2L1B3
**Non Infl-15**	19	2	63	f	currently	currently	never	never	never	never	never	none	A4L4+B3p
**Non Infl-16**	53	0	25	m	previously	never	currently	currently	never	never	never	sulfasalazine	A1L2B1
**Non Infl-17**	NA	0	23	m	never	previously	previously	currently	never	never	never	none	A2L3B1
**Non Infl-18**	22	0	38	f	previously	previously	previously	currently	never	previously	never	none	A1L34+B3p
**Non Infl-19**	NA	1	37	f	previously	previously	previously	currently	never	never	never	none	A2L3B3p
**Non Infl-20**	23	1	41	f	previously	never	previously	previously	never	currently	never	none	A2L4+B3p
**Non Infl-21**	7	NA	32	f	previously	never	previously	previously	never	currently	never	none	A2L4+B3p
**Non Infl-22**	159	3	32	m	previously	previously	previously	previously	never	currently	never	none	A2L3B1
**Non Infl-23**	NA	NA	25	f	never	never	previously	previously	never	previously	currently	none	
**Non Infl-24**	NA	NA	30	f	never	never	never	currently	never	currently	never	none	
**Non Infl-25**	21	2	53	f	previously	previously	previously	previously	never	currently	never	none	A2L4B3
**Non Infl-26**	NA	0	29	f	previously	previously	previously	previously	never	previously	never	none	A2L2B1
**Non Infl-27**	NA	4	19	m	previously	previously	previously	previously	never	currently	never	none	A2L3B3
**Non Infl-28**	82	0	49	m	previously	currently	previously	previously	never	currently	never	none	A2L1B3
