# Supplementary material for: High-dimensional profiling reveals Tc17 cell enrichment in active Crohn’s disease and identifies a potentially targetable signature
Source: Nat Commun. 2022 Jun 27;13:3688. doi: 10.1038/s41467-022-31229-z (PMC9237103; doi:10.1038/s41467-022-31229-z)
Supplement: Supplementary file 2 — Reporting Summary [file 41467_2022_31229_MOESM2_ESM.pdf]

nature portfolio

Corresponding author(s): Bertram Bengsch

Last updated by author(s): 17-April-2022

Reporting Summary

Nature Portfolio wishes to improve the reproducibility of the work that we publish. This form provides structure for consistency and transparency in reporting. For further information on Nature Portfolio policies, see our Editorial Policies and the Editorial Policy Checklist.

Statistics

For all statistical analyses, confirm that the following items are present in the figure legend, table legend, main text, or Methods section.

n/a	Confirmed	
☐	☒	The exact sample size (n) for each experimental group/condition, given as a discrete number and unit of measurement
☐	☒	A statement on whether measurements were taken from distinct samples or whether the same sample was measured repeatedly
☐	☒	The statistical test(s) used AND whether they are one- or two-sided<br>*Only common tests should be described solely by name; describe more complex techniques in the Methods section.*
☒	☐	A description of all covariates tested
☐	☒	A description of any assumptions or corrections, such as tests of normality and adjustment for multiple comparisons
☐	☒	A full description of the statistical parameters including central tendency (e.g. means) or other basic estimates (e.g. regression coefficient) AND variation (e.g. standard deviation) or associated estimates of uncertainty (e.g. confidence intervals)
☐	☒	For null hypothesis testing, the test statistic (e.g. F, t, r) with confidence intervals, effect sizes, degrees of freedom and P value noted<br>*Give P values as exact values whenever suitable.*
☒	☐	For Bayesian analysis, information on the choice of priors and Markov chain Monte Carlo settings
☒	☐	For hierarchical and complex designs, identification of the appropriate level for tests and full reporting of outcomes
☒	☐	Estimates of effect sizes (e.g. Cohen's d, Pearson's r), indicating how they were calculated

Our web collection on statistics for biologists contains articles on many of the points above.

Software and code

Policy information about availability of computer code

Data collection	Flowcytometric analysis was performed with a CytoFLEX (Beckman Coulter, Germany).
Data analysis	FlowJo software v10.6 (FlowJo LLC, USA) was used to analyze flow cytometric and mass cytometric data. Statistical analysis was performed using GraphPad version 8 (Prism Software Inc., USA) and R version 3.6 (https://www.r-project.org) respectively. Essential R packages used are: SummarizedExperiment, CATALYST, flowCore, ComplexHeatmap, limma, lme4, edgeR, FlowSOM, tsne, nlme, MASS, Rtsne. The R scripts used are available upon reasonable request.

For manuscripts utilizing custom algorithms or software that are central to the research but not yet described in published literature, software must be made available to editors and reviewers. We strongly encourage code deposition in a community repository (e.g. GitHub). See the Nature Portfolio guidelines for submitting code & software for further information.

Data

Policy information about availability of data

All manuscripts must include a data availability statement. This statement should provide the following information, where applicable:

- Accession codes, unique identifiers, or web links for publicly available datasets
- A description of any restrictions on data availability
- For clinical datasets or third party data, please ensure that the statement adheres to our policy

Mass cytometry data will be made available on Flowrepository.org

Some analyses in this manuscript are based on the previously published transcriptomic dataset E-MTAB-331 (https://www.ebi.ac.uk/arrayexpress/experiments/E-MTAB-331/)

Field-specific reporting

Please select the one below that is the best fit for your research. If you are not sure, read the appropriate sections before making your selection.

[x] Life sciences   [ ] Behavioural & social sciences   [ ] Ecological, evolutionary & environmental sciences

For a reference copy of the document with all sections, see nature.com/documents/nr-reporting-summary-flat.pdf

Life sciences study design

All studies must disclose on these points even when the disclosure is negative.

Sample size	61 patients with Crohn's disease and 25 healthy donors were included in the study (Table 1 and Supplementary Table 2-4). Sample size was chosen based on previous experience in the lab and is similar to published studies (Globig et al, 2014).
Data exclusions	no data were excluded.
Replication	all flow cytometry experiments were performed in multiple individual experiments (n=/>3) and effects were reproduced between the repeats. mass cytometry data were acquired in one batch and findings were confirmed using flow cytometry.
Randomization	not applicable, patients were assigned to "active disease" and "inactive disease" based on clinical parameters. no interventional treatment in patients was performed in this study.
Blinding	not applicable, patients were assigned to "active disease" and "inactive disease" based on clinical parameters.

Reporting for specific materials, systems and methods

We require information from authors about some types of materials, experimental systems and methods used in many studies. Here, indicate whether each material, system or method listed is relevant to your study. If you are not sure if a list item applies to your research, read the appropriate section before selecting a response.

Materials & experimental systems

n/a	Involved in the study	
[ ]	[x]	Antibodies
[x]	[ ]	Eukaryotic cell lines
[x]	[ ]	Palaeontology and archaeology
[x]	[ ]	Animals and other organisms
[ ]	[x]	Human research participants
[x]	[ ]	Clinical data
[x]	[ ]	Dual use research of concern

Methods

n/a	Involved in the study	
[x]	[ ]	ChIP-seq
[ ]	[x]	Flow cytometry
[x]	[ ]	MRI-based neuroimaging

Antibodies

Antibodies used	CD8 BV650 (RPA-T8, BioLegend, #301042,validated by manufacturer in hPBMC, 1:800), PD1 BV786 (EH12.1, BD Biosciences, #563789, validated by manufacturer in hPBMC, 1:100), PD1 BV421 (EH12.2H7, BioLegend, #329920, validated by manufacturer in hPBMC, 1:100), PD1 PerCP-eFluor710 (eBioJ105, eBioscience, #46-2799, validated by manufacturer in hPBMC, 1:50), CD26 FITC (2A6, eBioscience, #11-0269, validated by manufacturer in hPBMC, 1:100), CD6 PE (BL-CD6, BioLegend, #313906, validated by manufacturer in hPBMC, 1:400), CD27 PE-Dazzle594 (M-T271, BioLegend, #356422, validated by manufacturer in hPBMC, 1:400), CD39 PerCP-eFluor710 (eBioA1, eBioscience, #46-0399-42, validated by manufacturer in hPBMC, 1:200), CD69 PE-Cy7 (FN50, eBioscience, #25-0699, validated by manufacturer in hPBMC, 1:50), CD161 APC (191B8, Miltenyi, #130-092-678, validated by manufacturer in hPBMC, 1:50), IFN-γ APC-eFluor780 (4S.B3, eBioscience, #47-7319, validated by manufacturer in hPBMC, 1:800), Fixable viability dye BV510 (eBioscience, #65-0866-14, 1:2000), Fixable viability dye APC-eFluor780 (eBioscience, #65-0865-14, 1:5000), IL-17 BV605 (BL168, BioLegend, #512326, validated by manufacturer in hPBMC, 1:100), IL-17F BV786 (O33-782, BD Biosciences, #564265, validated by manufacturer in hPBMC, 1:100), RORγt PE (# 600380, R&D Systems, #IC6006P-100, validated by manufacturer in hPBMC, 1:100), CD56 BV650 (5.1H11, BioLegend, #362532, validated by manufacturer in hPBMC, 1:100), TCRαβ AlexaFluor488 (IP26, BioLegend, #306712, validated by manufacturer in hPBMC, 1:100), TCRαβ BV421 (IP26, BioLegend, #306722, validated by manufacturer in hPBMC, 1:100), TCRγδ PE (5A6.E9, Life Technologies, #564157, validated by manufacturer in hPBMC, 1:100), CD8 PE-Dazzle594 (RPA-T8, BioLegend, #301057, validated by manufacturer in hPBMC, 1:800), TCR Vα7.2 PE-Cy7 (3C10, BioLegend, #351711, validated by manufacturer in hPBMC, 1:25), TCR Vα7.2 FITC (3C10, BioLegend, #351704, validated by manufacturer in hPBMC, 1:100), TCR Vα24Jα18 PE-Cy7 (6B11, Invitrogen, #25-5806-42, validated by manufacturer in hPBMC, 1:100), CD3 AlexaFluor700 (SK7, BioLegend, #344822, validated by manufacturer in hPBMC, 1:800), IL-17 PE (eBio64DEC17, eBioscience, #12-7179-42, validated by manufacturer in hPBMC, 1:200), TNF PE-Cy7 (MAb11, BioLegend, #502930, validated by manufacturer in hPBMC, 1:800), pSTAT3 Alexa 647 (4/P-STAT3, BD Biosciences, #562071, validated by manufacturer in hPBMC; 1:50), CD3 PerCP (SK7, BD Biosciences, #345766, validated by manufacturer in hPBMC, 1:200), CD8 Krome Orange (B9.11, Beckman Coulter, B00067, validated by manufacturer in hPBMC,1:200), CD45RA PeCy7 (HI100, BD Biosciences, 337186, validated by manufacturer in hPBMC,

1:200), Isotype IgG2a κ Alexa 647 (MOPC-173, BD Biosciences, #558053, validated by manufacturer, 1:50), CD4 BV786 (L200, BD Biosciences, #563914, validated by manufacturer in hPBMC, 1:200), CD4 BV421 (RPA-T4, BioLegend, #300532, validated by manufacturer in hPBMC, 1:100). Itolizumab (Alzumab™, clone CD6D1, Biocon, India, 40 µg/ml). anti-CD6 antibody, clone UMCD6 (Sigma Aldrich, Germany, MABF2105, validated by manufacturer, 10 µg/ml). Isotype human IgG1 κ (QA16A12, BioLegend, #403502, validated by manufacturer, 40 µg/ml), Isotype mouse IgG1 κ (MG1-45, BioLegend, #401408, validated by manufacturer, 10 µg/ml).
CD45, clone HI30, Fluidigm, #3089003B, validated by manufacturer in hPBMC, 1:300; CD6, clone MT605, BD, #555356, validated by manufacturer in hPBMC, 1:100; CD57, clone TB01, Ebioscience, #MA5-16948, validated by manufacturer in hPBMC, 1:200; CD3, clone UCHT1, Biolegend, #300402, validated by manufacturer in hPBMC, 1:200; IFN-G, clone B27, Biolegend, #506501, validated by manufacturer in hPBMC, 1:300; CD4, clone RPA-T4, Biolegend, #300502, validated by manufacturer in hPBMC, 1:200; CTLA-4, clone BNI3, BD, #555851, validated by manufacturer in hPBMC, 1:100; TNF, clone MAb11, Ebioscience, #14-7349-81, validated by manufacturer in hPBMC, 1:400; CD8, clone RPA-T8, Biolegend, #301002, validated by manufacturer in hPBMC, 1:300; CD45RA, clone H100, BD, #555486, validated by manufacturer in hPBMC, 1:100; CD69, clone FN50, Biolegend, #310902, validated by manufacturer in hPBMC, 1:100; CCL3, clone MAB2701, R&D, #MAB2701-100, validated by manufacturer in hPBMC, 1:150; IL-22, clone 22URTI, Fluidigm, #3150007B, validated by manufacturer in hPBMC, 1:100; CD39, clone A1, Biolegend, #328221, validated by manufacturer in hPBMC, 1:100; IL-2, clone MQ1-17H12, Ebioscience, #14-7029-81, validated by manufacturer in hPBMC, 1:150; TIM-3, clone F38-2E2, Fluidigm, #3153008B, validated by manufacturer in hPBMC, 1:200; XCL-1, clone 109001, R&D, #MAB6951, validated by manufacturer in hPBMC, 1:200; CD27, clone L128, Fluidigm, #3155001B, validated by manufacturer in hPBMC, 1:200; Helios, clone 22F6, Biolegend, #137202, validated by manufacturer in hPBMC, 1:200; PD-1, clone EH12.2H7, Biolegend, #329902, validated by manufacturer in hPBMC, 1:100; GM-CSF, clone BVD2-21C11, Fluidigm, #3159008B, validated by manufacturer in hPBMC, 1:100; Tbet, clone 4B10, Fluidigm, #3160010B, validated by manufacturer in hPBMC, 1:200; CRTAM, clone Cr24.1, Biolegend, #339102, validated by manufacturer in hPBMC,, 1:200; IL-21, clone 3A3-N2, Biolegend, #513001, validated by manufacturer in hPBMC, 1:200; CXCR3, clone G025H7, Biolegend, #353702, validated by manufacturer in hPBMC, 1:200; LIFR, clone 1C7, EMD, #MABD150, validated by manufacturer in hPBMC, 1:100; Eomes, clone WD1928, Ebioscience, #14-4877-82, validated by manufacturer in hPBMC, 1:200; Areg, polyclonal, Thermo, #RB-257-PABX, validated by manufacturer in hPBMC, 1:200; CD38, clone HIT2, Fluidigm, #3167001B, validated by manufacturer in hPBMC,1:200; Tox, clone Rea473, Miltenyi, custom, validated by manufacturer in hPBMC, 1:100; Il-13, clone JES10-5A2, Fluidigm, #3169016B, validated by manufacturer in hPBMC, 1:100; CXCR5, clone RF8B2, BD, #552032, validated by manufacturer in hPBMC, 1:100; CADM1, polyclonal, EMD, #ABT66, validated by manufacturer in hPBMC 1:50; IL-17A, clone BL168, Fluidigm, #3172020B, validated by manufacturer in hPBMC, 1:200; CD120B, clone hTNFR-M1, Biolegend, #551311, validated by manufacturer in hPBMC, 1:100; IL-10, clone JES3-12GB, Biolegend, #501505, validated by manufacturer in hPBMC, 1:100; OSM, clone 17022, R&D, #MAB2951, validated by manufacturer in hPBMC, 1:100; LIF, clone M1506B09, Biolegend, #674702, validated by manufacturer in hPBMC, 1:100; CD16, clone 3G8, Fluidigm, #3209002B, validated by manufacturer in hPBMC, 1:200.

Validation

Custom conjugated antibodies were validated at several levels, starting from assessment of expected protein concentration after conjugation until they were titrated and tested for optimal signal/noise ratio based on expression patterns on cell populations that served as a negative and positive controls. We also validated mass cytometry antibodies in addition to this strategy with flow cytometry staining. Anti CD6 antibodies were validated by flow cytometric analysis of CD6 expression after 15min incubation of PBMC with the antibodies.

Human research participants

Policy information about studies involving human research participants

Population characteristics

CD patients were recruited at the IBD outpatient unit and endoscopy unit of the University Hospital of Freiburg. Additional CD samples were obtained from the biobank of the Immunology in IBD Initiative (I3) at the Hospital of the University of Pennsylvania. Following patients' informed consent, peripheral blood, intestinal biopsies and clinical parameters including laboratory data were collected. 61 patients with Crohn's disease and 25 healthy donors were included in the study (Table 1 and Supplementary Table 2-4).

Recruitment

CD patients were prospectively and continuously recruited at the IBD outpatient unit and endoscopy unit of the University Hospital of Freiburg. Additional CD samples were obtained from the biobank of the Immunology in IBD Initiative (I3) at the Hospital of the University of Pennsylvania.

Ethics oversight

Study participants were recruited with approval of the Institutional Review Boards (Ethics committee of the Albert-Ludwigs-University, Freiburg, #407/16 & #14/17, University of Pennsylvania Institutional Review Board #814428). The study was performed in agreement with the principles expressed in the Declaration of Helsinki (2013). Written informed consent was received from participants prior to inclusion in the study.

Note that full information on the approval of the study protocol must also be provided in the manuscript.

Flow Cytometry

Plots

Confirm that:

- [x] The axis labels state the marker and fluorochrome used (e.g. CD4-FITC).
- [x] The axis scales are clearly visible. Include numbers along axes only for bottom left plot of group (a 'group' is an analysis of identical markers).
- [x] All plots are contour plots with outliers or pseudocolor plots.
- [x] A numerical value for number of cells or percentage (with statistics) is provided.

Methodology

Sample preparation

Briefly, isolation of peripheral blood mononuclear cells (PBMC) was done using a ficoll-histopaque density gradient centrifugation (Ficoll Paque PLUS, GE Healthcare Life Sciences). Blood drawn into EDTA tubes was diluted 1:1 with PBS, subsequently overlaid 2:1 on ficoll, and centrifuged for 20 min (800g, acceleration/ brake 0, room temperature (RT)). The collected buffy coat was washed in phosphate buffered saline (PBS, (Corning, #21-040-CV) and pelleted prior to further use (10 min, 500g, acceleration/brake 9, RT).

Lymphocytes from intestinal biopsies isolated at the University Medical Center Freiburg used for flow cytometric analyses were mechanically homogenized through a 70 µm cell strainer (ThermoFisher, #08-771-2) before washing and biobanking (not subjected to an enzymatic digestion). Samples were obtained during ileocolonoscopy and origin grouped into ileum/ colon/sigma. 34.6% of biopsies were from the colon, 34.6% were taken from the ileum and 30.7% from the sigma. Samples were processed and frozen at the day of sample collection and thawed on the day of the respective experiment. For flow cytometry studies this approach was chosen to maximize cell yield and staining performance of chemokine receptors.

For mass cytometry experiments, lamina propria lymphocytes (LPL) and intraepithelial lymphocytes (IEL) were isolated using enzymatic digestion per I3 biobank SOPs. Freeze media (FM: 90% FBS (Gem Cell, 100-500), 10% DMSO (Sigma, D2650-5X10ML), DNase I (Sigma, #D5025-150KU, prepared stock of 4mg/ml) and Collagenase/Dispase (Roche, 500mg, #11097113001, prepared stock of 50mg/ml) were brought to RT. Biopsies were placed into a 50 ml Falcon tube containing 3 ml epithelial strip buffer (1X PBS, 5 mM EDTA (ThermoFisher, #15575020), 1 mM DTT (ThermoFisher, #R0861), 5% FBS, 1% penicillin/streptomycin (Gibco, #15140-122) and incubated for 10 minutes in a 37°C water bath. After vortexing, supernatant was transferred into an Eppendorf tube and spun down (17000 x g, 10 min, 4°C, accuSpin Micro 17 centrifuge). Supernatant was removed and remaining tissue was further digested in 5 ml of wash buffer (WB: RPMI-1640 (Corning Life Sciences, #10-040-CV), 2% FBS, 1% L-Glutamine (Lonza, #17-605E), 1% pen/strep) supplemented with 50 µl of DNase I and 25 µl of Collagenase/Dispase stocks for 20 minutes at 37°C. Following vortexing, the sample was gently strained through a 70 µm strainer and LPL were washed off with 20 ml of WB. Cells were spun down (5 min, 800 x g, 4°C), cryopreserved as described above and thawed at the day of the respective experiment. CyTOF acquisition was performed using LPL samples.

Instrument

Flowcytometric analysis was performed with a CytoFLEX (Beckman Coulter, Germany)

Software

FlowJo software v10.6 (FlowJo LLC, USA) was used to analyze flow cytometric and mass cytometric data

Cell population abundance

Cell population abundance is indicated in the figures.

Gating strategy

please see gating strategy in the Supplementary Information; in brief: Lymphocytes --> singlets--> live cells --> CD3 T cells --> CD8 --> individual markers

☒ Tick this box to confirm that a figure exemplifying the gating strategy is provided in the Supplementary Information.
